# The Effect on the Transcriptome of *Anemone coronaria* following Infection with Rust (*Tranzschelia discolor*)

**DOI:** 10.1371/journal.pone.0118565

**Published:** 2015-03-13

**Authors:** Marina Laura, Cristina Borghi, Valentina Bobbio, Andrea Allavena

**Affiliations:** CRA—Unità di Ricerca per la Floricoltura e le Specie Ornamentali, Corso Inglesi 508, 18038 Sanremo (IM), Italy; Louisiana State University, UNITED STATES

## Abstract

In order to understand plant/pathogen interaction, the transcriptome of uninfected (1S) and infected (2I) plant was sequenced at 3’end by the GS FLX 454 platform. *De novo* assembly of high-quality reads generated 27,231 contigs leaving 37,191 singletons in the 1S and 38,393 in the 2I libraries. ESTcalc tool suggested that 71% of the transcriptome had been captured, with 99% of the genes present being represented by at least one read. Unigene annotation showed that 50.5% of the predicted translation products shared significant homology with protein sequences in GenBank. In all 253 differential transcript abundance (DTAs) were in higher abundance and 52 in lower abundance in the 2I library. 128 higher abundance DTA genes were of fungal origin and 49 were clearly plant sequences. A tBLASTn-based search of the sequences using as query the full length predicted polypeptide product of 50 *R* genes identified 16 *R* gene products. Only one *R* gene (*PGIP*) was up-regulated. The response of the plant to fungal invasion included the up-regulation of several pathogenesis related protein (PR) genes involved in JA signaling and other genes associated with defense response and down regulation of cell wall associated genes, non-race-specific disease resistance1 (*NDR1*) and other genes like *myb*, *presqualene diphosphate phosphatase* (*PSDPase*), a *UDP-glycosyltransferase 74E2-like* (*UGT*). The DTA genes identified here should provide a basis for understanding the *A*. *coronaria/T*. *discolor* interaction and leads for biotechnology-based disease resistance breeding.

## Introduction

The genus *Anemone* (*Ranunculaceae*) harbor over 120 species, distributed over the temperate zones of both hemispheres; many of these species are cultivated as ornamentals. The poppy anemone (*Anemone coronaria*), of Mediterranean origin, is the progenitor of most of the cut flower, pot and garden plant varieties currently cultivated [1]. In nature, seeds produced in late spring usually germinate in autumn and offspring starts flowering the following year. Under cultivation practice, rhizomes, derived from seed by one growth cycle in the nursery, are planted by growers, after vernalization, in order to shorten the time from planting to harvest.

A major biotic constraint for *Anemone* producers is the rust disease, caused by the basidiomycete *Tranzschelia discolor* [2], which has become an aggressive pathogen in recent years, following the widespread exploitation of tetraploid cultivars of *A*. *coronaria*. The pathogen infects *Prunus* spp. as its primary host and some members of *Ranuncolaceae* as its alternate host. During the production of *A*. *coronaria* rhizomes, seedlings frequently are infected by inoculum which has developed on *Prunus* spp. foliage, although the infected plants remain asymptomatic until the following vegetative cycle. The disease has a major impact on flower yield and quality and finally plants became rusted and dies.

The breeding of resistant varieties of *A*. *coronaria* has been hampered by poor state of knowledge regarding the host/pathogen interaction.

Rust pathogen fungi are obligate biotrophic parasites [3]. A successful infection requires that effectors, coded by avirulence (*Avr*) genes, are secreted into infected tissues to repress and manipulate host defense [4]. In turn, plants possess several hundreds of resistance (*R*) genes that trigger strong defense responses [5]. The ability of pathogen effectors to manipulate host functions and escape R protein recognition is thought to be the key of compatibility [6]. Specific recognition is thought to be mediated by ligand receptor binding [7]. In order to survive, plants have engaged a co-evolutionary battle engendering a wide range of constitutive and inducible defenses [8]. Constitutive defenses include many preformed barriers such as cell walls, waxy epidermal cuticle, tricomes and bark. In addition, plants have developed two innate immune systems for defense [8,9]. The primary innate immunity, is driven by pattern recognition receptor (PRRs), that recognize microbe-associated molecular patterns (MAMPs) and triggers primary defense responses, such as cell wall alterations, deposition of callose and the accumulation of defense-related proteins including chitinases, glucanases and proteases [5,9]. Virulent pathogen are able to suppress basal defense activated in the primary innate immune system, developing mechanisms to escape recognition of MAMPs [8,10]. Therefore plants have developed a secondary defense response through resistance proteins (RPs) that monitor the effectors or their perturbations of host targets and often culminate in a hypersensitive response (HR). The hypersensitive response is characterized by localized cell and tissue death at the site of infection [11]. This strong defense reaction is characterized by the accumulation of reactive oxygen species (ROS), antimicrobial proteins and phytoalexins that lead to a local cellular suicide, which stops biotrophic pathogens from further growth. Plants are also protected by a mechanism called systemic acquired resistance (SAR), which is induced simultaneously with local primary and secondary immune response [12], providing durable protection against challenge infection by a broad range of pathogens [13–15]and is dependent on different plant hormones, as salicylic acid (SA), jasmonic acid (JA), ethylene (ET), abscisic acid (ABA). Next-generation sequencing (NGS) technology has revolutionized the acquisition of nucleic acid sequence and made major contributions to our understanding of genome structure, gene expression and regulation [16–18]. The RNA-Seq platform provides a direct count of the number of specific transcripts present in an mRNA sample and thus gives an informative means not only of acquiring transcriptomic sequence, but also of identifying differential transcription [19]. The accuracy of its measurement of transcript abundance is as high, if not higher than is possible using microarray technology [20–22]. An RNA-Seq variant, that consider sequencing of 3’ ends only, permit detection of rare transcript even in case of a low number of reads [23]. As a result, the approach has been widely employed to study transcription in fungal, plant and animal genomes [24,25]. The NGS FLX 454 pyrosequencing technology (Roche, Brandford, CT, USA) has been widely used for de novo sequencing and analysis of trascriptomes in non-model organisms, such as olive [26], chestnut [27], *Artemisia annua* [28], ginseng [29], blueberry [30] bracken fern [31] and in switchgrass [32].

Here, we report the use of FLX 454 technology to analyze the transcriptome of *A*. *coronaria* and in particular to determine what change in transcription are induced when plant is infected by *T*. *discolor*.

## Materials and Methods

### RNA extraction

**Fig 1:**
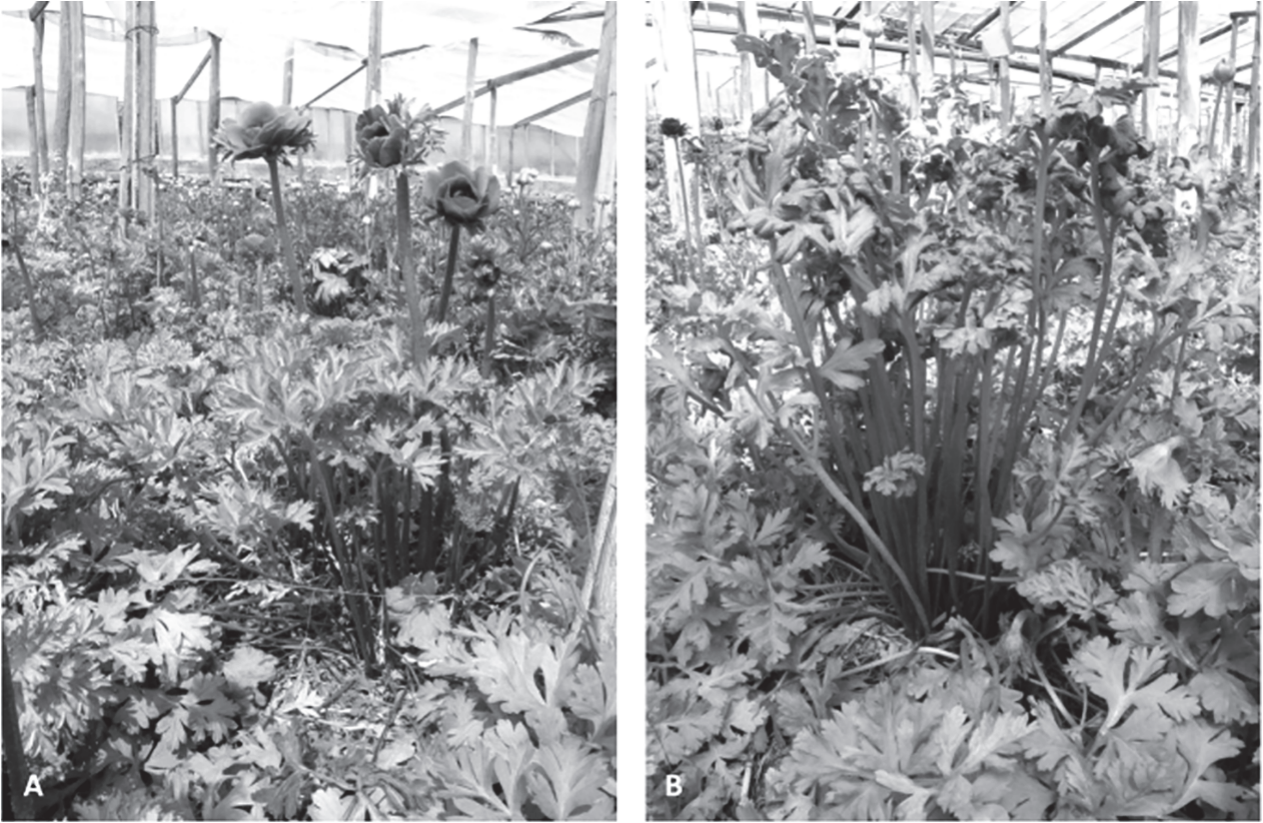
Healthy and *Tranzschelia discolor* infected plant. In comparison to healthy plant (A), during biotropich relationship, infected plant displays plethoric vegetation with robust leaf stems and curly leaf lamina. Flowering is strongly repressed.

*A*. *coronaria* plants (cv ‘Tetraelite’ blue) were grown under shade netting. Thirty healthy and thirty *T*. *discolor* infected plants were monitored throughout their life cycle for disease symptoms. Early infected plants were easily identified by plethoric vegetation, robust, erect leaf stems and thick, slightly curled leaf lamina. Leaves of infected plants were harvested as soon as plant showed disease symptoms. This time point covers leaf invasion by hyphae from the plant rhizomes under real field condition. Healthy leaves of the same age were harvested from uninfected plants (Fig. 1). Leaf tissues was snap-frozen in liquid nitrogen and stored at -80°C until required. Total RNA was isolated from 100 mg of frozen leaf using an RNeasy Plant Mini kit (QIAGEN GmbH, Hilden, Germany) and treated with recombinant DNase I (QIAGEN) within the column, following the manufacturer’s protocol. The concentration of recovered RNA was estimated using a Nanodrop 2000 device (Thermo Fisher Scientific Inc., Wilmington, DE, U.S.A.) and its integrity assessed using a Total RNA Stdsens chip (Experion system, Biorad, Hercules, CA, USA). High quality RNAs from five uninfected plants were combined to form the “1S” pool and similarly from five infected ones to form the “2I” pool.

### 454 titanium sequencing

Ten μg of RNA of each pool were sent to Eurofins MWG-Operon (Ebersberg, Germany – http://www.eurofinsdna.com/home.html) for preparation of two 3’ c-DNA libraries (“1S” and “2I”) and sequencing using GS FLX 454 Titanium system (454 Life Sciences, a Roche company, Branford, CT, USA).

### Assembling, annotation and functional analysis

The Roche 454 high quality (HQ) reads generated in this study were deposited in the NCBI Sequence Read Archive (http://www.ncbi.nlm.nih.gov/sra) under accession SRX447797. After trimming of adapter sequences and removing reads shorter than 40 nt, libraries were mass assembled into a set of transcript contigs using CLC Genomics Workbench 5.0 operating with its default minimum identity setting of 0.8. Unigenes (contigs and remaining unique singletons) were annotated using a BLASTx search of the NCBI non-redundant protein database (nr), with the help of Blast2GO v2.5 (http//:www.blast2go.org) applying an e-value threshold of 1e-3. Blast2GO was also used to obtain gene ontology (GO) information. Sequences were annotated with respect to GO term applying the default e-value of 1e-6 and the “Augment Annotation by ANNEX” function was used to refine annotation. An InterPro search was performed for the sequences which were unsuccessfully annotated by BLASTx analysis [33]. The Plant GO-Slim algoritm was used to assign GO terms. Pathway assignment was processed with KEGG database. To identify GO categories represented differentially between the two libraries, an enrichment analysis was performed using two tail Fisher’s exact test as implemented in Blast2GO, applying a False Discovery Rate (FDR) of 0.05 and the Benjamini and Hochberg [34] Multiple Testing Correction; for this purpose, the annotated sequences of the library generated from 2I pool (contigs and singletons) was used as the test set and those of the library from the 1S pool as references set [35]. To identify genes involved in the disease response, the predicted unigene products were queried using BLASTx (as implemented in the CLC Genomics Workbench 5.0 with the default parameters) using the peptide sequences of 50 known disease resistance (*R*) gene [36], covering each the five major *R* gene classes.

### Assessment of transcriptome coverage

Coverage of the transcriptome was estimated using the web-based ESTcalc tool [37]. The number of reads (610,561), the average read length of 330 nt and one run for 454 GS FLX technology determined the input parameters.

The number of eukaryotic ultraconserved orthologs (UCOs) represented in the dataset was obtained from a tBLASTx query based on the 357 *Arabidopsis thaliana* UCOs available at http://compgenomics.ucdavis.edu/compositae_reference.php; the chosen e-value threshold was 1e-10.

### Analysis of differential transcript abundance (DTAs)

DESeq package [38] was chosen to identify gene with DTA. It integrates several statistical methods, that can estimate a theoretical replicate when an experimental one is not provided and has been routinely used [39,40].

The number of reads contributing to each contig was compared for each gene of the 1S and 2I libraries. The FDR threshold was set at 0.05.

### Phylogenetic analysis and alignment of DTA genes

The selected unigenes for the alignment and phylogenetic analysis were blasted against the NCBI nr protein database, using a BLASTx search (http://blast.ncbi.nlm.nih.gov/blast/). The full length amino acid sequences with higher "Max score" and "Identity" percentage were selected for analysis.

Clustal Omega program (http://www.ebi.ac.uk/Tools/msa/clustalo/) was used to obtain sequences alignment (Gonnet matrix). The maximum likelihood (ML) method from the MEGA program (version 6.06) [41] was used to create phylogenetic trees of selected DTA genes. The reliability of each branch was tested by bootstrap analysis with 100 replications.

### Validation of DTAs using qPCR

Ten plant DTAs genes (five up- and five down-regulated) putatively involved in the response to *T*. *discolor* infection, along with three fungal genes, were subjected to RT-PCR and qPCR analysis as described by Laura et al. [42]. The templates compared were the pools of uninfected and infected plants analyzed by pyrosequencing (sample A). Primers were designed using Primer 3 plus software (http://primer3plus.com/cgi-bin/dev/primer3plus.cgi) and the *A*. *coronaria 18S rRNA* gene was employed as the reference (Table 1). For each gene, three biological replicates and three technical replicates were performed. Two additional sample (B and C) each composed of five of uninfected and infected plants, not included in the libraries, were analyzed further.

**Table 1:** Primer sequences of differential transcript abundance (DTA) genes and *Tranzschelia discolor* genes used for qPCR analisys.

Contig	Sequence	Origin	Fwd primer (5’-3’)	Rev primer (5’-3’)
2476	18S Rrna (reference gene)	Plant	gagagagggggaagaaaagg	gtggaacagtgagggacaag
9288	NDR1-like	Plant	ccctaccaccgttgtttcac	cactctacccatcagcacca
4448	inner membrane protein PPF-1	Plant	atctgacgttgctgaagcct	gcaccaaaaatgagctaagcc
6057	late embryogenesis abundant	Plant	catccgttcttccagcgac	tgtgccgacttccataccc
9205	myb family transcription factor	Plant	tggcaatcaaggtcaacaac	cctcggtcttttcttcatcg
17626	XTH	Plant	tgatggctgtgaggagtctg	attctggtggcactgtaggg
3404	acidic chitinase	Plant	tggtagcactgtaggcctcg	tcttgatagccggcaaaact
12518	COMT	Plant	acttgtgaacctgctctgcc	actcttcctctgtgcgatgc
18538	metallothionein protein	Plant	cgtcaggctgtaactgtggt	tgctccaacacccatctctg
7408	PDF1.1	Plant	tgggacatggtcaggagtct	ggcggggaatttgtagttgc
21204	PGIP	Plant	cggtgatgcttcggttctgt	catttggtggtcaaggggg
21597	bax Inhibitor family protein	Fungi	ccctcgttttgcccgtca	cggcttcatcctaccttgttgc
21934	nucleoside diphosphate kinase	Fungi	cctttgcggtttgtggattg	atgtgctttgaaggggtgag
20893	inorganic phosphate transporter 1–7	Fungi	gcagcctctggaaaacttgg	gtcagcagtcccgtaaacatca

After normalization, transcript abundances were compared using the 2^−ΔΔCt^ method [43]. The Wilcoxon-Mann-Whitney [44] test was applied as implemented in the GraphPad InStat v3.10 package (www.graphpad.com). Transcript abundance data were expressed in the form mean ± standard error (SE).

## Results and Discussion

### EST sequencing and assembly

The GS FLX 454 output yielded 304,487 (1S library) and 306,074 (2I library) raw reads of average length, 330 and 322 nt, respectively. The size distribution of reads is given in S1 Fig. and a summary of sequencing and assembly outcomes presented in Table 2. Respectively, 12,052 and 12,488 sequences were discarded on the basis of shortness of length (< 40bp) or low quality score (Mira version 4.0.2), resulting in the acquisition of, respectively, 292,435 and 293,586 HQ reads. These HQ reads were assembled into 27,231 contigs leaving 37,191 singletons in the 1S library and 38,393 in the 2I library. The number of contigs specific to one library was 5,802 (1S library) and 9,085 (2I library). Among the contigs, their length varied from 40 to 1367 bp (mean 377 nt) and 507 were longer than 800 nt (S2 Fig.); the length of the singletons, ranged from 40–696 nt (mean 247.5 nt) in the 1S library and from 40–767 nt (mean 248 nt) in the 2I library. Sequencing coverage, as estimated from the mean number of reads per contig [45], was 19.52.

**Table 2:** Overview of the sequencing and assembly.

454 pyrosequencing terms	Sequences (n)		Bases (bp)	
	1S	2I	1S	2I
**Sequencing**				
High-quality (HQ) reads	304,487	306,074	100,541,607	98,494,613
Average sequence length of HQ reads	330.2 bp	321.8 bp	-	-
N° of reads used in assembling	292,435	293,586	92,327,408	90,081,755
**Contigs**				
Reads mass assembled in contigs	255,244	255,193	83,123,220	80,564,794
N° of contigs	27,231		10,262,491	
Average length	377 bp		-	-
Range of length	40–1367 bp		-	-
N50	443 bp		-	-
**Singleton**				
N° of singletons	37,191	38,393	9,204,188	9,516,961
Average length	247.5 bp	248 bp	-	-
Range of lenght	40–696 bp	40–767 bp	-	-
**Unigenes**	**64,422**	**65,624**	**19,466,679**	**19,779,452**

### Transcriptome coverage

Since the genomic sequence of the *A*. *coronaria* is unavailable, the true size and composition of its transcriptome is unknown. Thus the simulation-based ESTcalc tool [37], was used to estimate the coverage of the transcriptome produced by the RNA-Seq data set. This exercise suggested that 71% of the transcriptome had been captured, with 99% of the genes present being represented by at least one read (Table 3). With respect to the UCOs, 348 of the 357 tested sequences were represented in the *A*. *coronaria* contigs.

**Table 3:** EST calc-based transcriptome coverage.

**Input parameters**	**EST calc**
Number of technologies	1
Technology	454 GSFLX
Library type	non-normalized
Number of Plates	0.5
Reads/Plate	610,561
Mean read lenght (bp)	330
**Predicted assembly**	**EST calc**
Total Sequence Amount (MB)	100.7
Total Assembled Sequence (MB)	21.1
Unigene count	37,822
Mean unigene length (bp)	558
Mean unigene length (longest unigene per gene, bp)	812
Singleton yield (%)	33
Percent transcriptome (%)	71
Percent of genes tagged (%)	99
Percent of genes with 90% coverage (%)	41.6
Percent of genes with 90% coverage by largest unigene (%)	29.7
Percent of genes with 100% coverage (%)	9.7
Percent of genes with 100% coverage by largest unigene (%)	9

### Sequence annotation

Unigenes annotation, through use of the Blast2GO tool, showed that 50.5% of the predicted translation products shared significant homology with known protein sequences deposited in GenBank and 1.7% with hypothetical proteins, leaving 47.8% of the sequences unannotated. The proportion of sequences lacking any BLASTx alignment and shorter than 250 nt was 42.1% (S3 Fig.). Short sequences are thought to derive predominantly from the highly divergent 3’ untranslated regions (UTRs), so may account for the high proportion of the low homology sequences. An InterPro search of 13,028 unannotated contigs identified 5,891 as harbouring known protein domains.

The BLASTx positive contigs identified *Vitis vinifera* (grape) as the most frequently occurring species, followed by *Populus tricocarpa* (black cottonwood), *Ricinus communis* (the castor oil plant), *Glycine max* (soybean) and *Puccinia graminis* (cereal stem black rust) (S4 Fig.). The number of fungal contigs identified was 1203 (S2 Table) of which 1194 were not represented in the 1S library. The presence of eight fungal contigs (11 reads) in the 1S library is thought to reflect field-based aeciospores contamination. None of the 1S library singletons matched sequences in the *P*. *graminis* proteome.

### Gene Ontology (GO) annotation

Sequences showing significant similarity to previously annotated proteins were assigned GO terms based on their associated biological processes (P), molecular functions (F) and cellular components (C). Plant specific GO slim terms were associated with 10,362 (38%) of the contigs, of which 8,433 were given an F, 6,726 a C and 6,451 a P assignation. The GO categories represented showed no significant bias and were distributed similarly to what has been described in other plant species [31,46,47].

**Fig 2:**
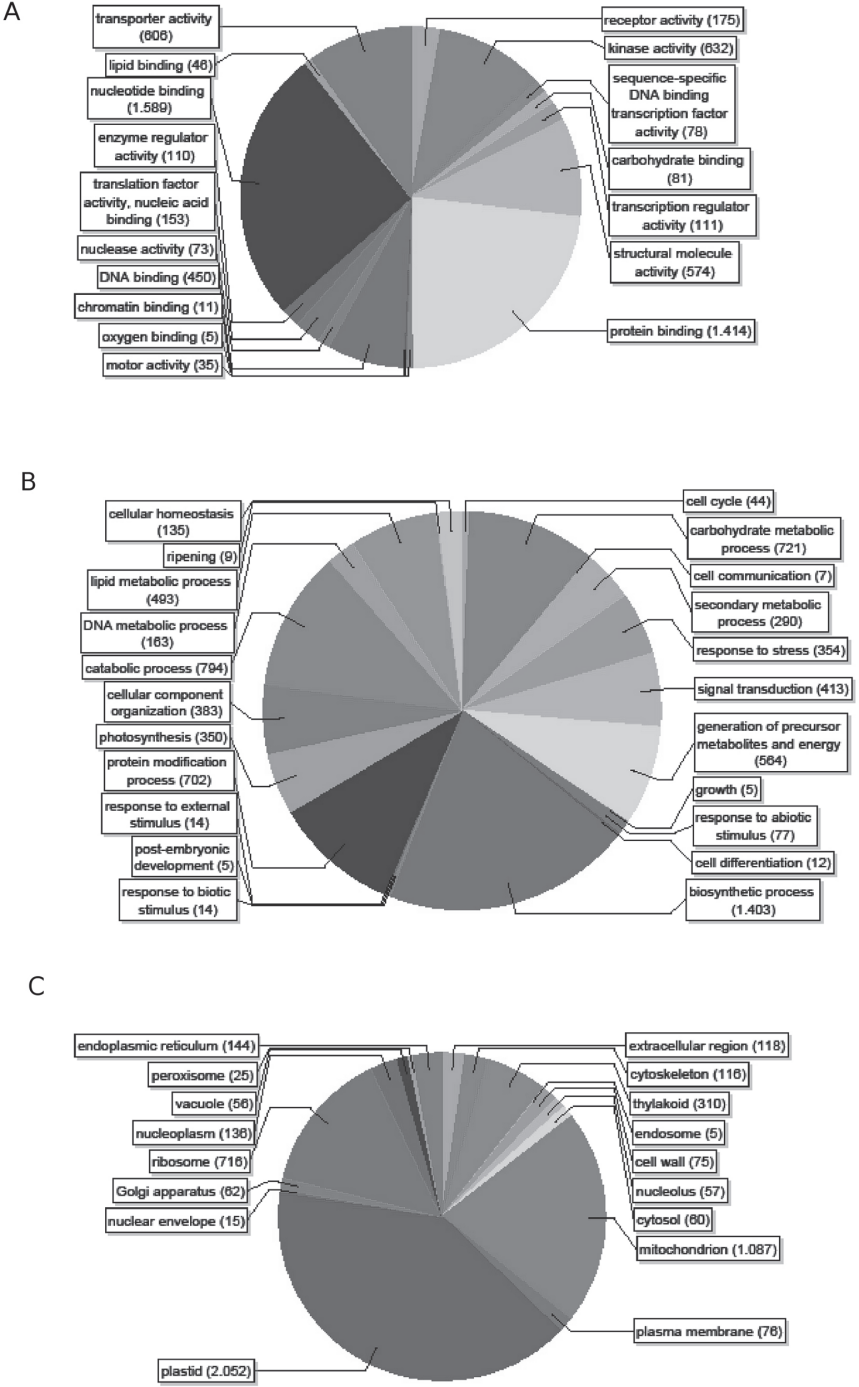
GO multilevel-Pie. Pie chart representation of Gene Ontology classification of (A) biological process (B) cellular component (C) molecular function, using a sequences cutoff of 5.0.

The Predominant P categories were biosynthetic process, catabolic process, carbohydrate metabolic process and protein modification process (Fig. 2A). The C assignation of most of the contigs was to the plastids or mitochondrion, but many were associated with the ribosome (Fig. 2B). Nucleotide binding, protein binding, kinase activity and transporter activity were the major F categories present. Genes involved in the responses to stress (354), abiotic (14) and biotic stimulus (77) and signal transduction (413) are also well represented (Fig. 2C).

Fisher’s Exact test confirmed that the distribution of GO categories differed between the two libraries. Specifically, 447 GO categories were differentially represented (S1 Table), of which 304 involved P, 67 F and 76 C. In all, 267 GO categories were under-represented in the 2I library and 180 over-represented. Among the latter, were genes encoding transferase activity (GO:0016740), hydrolase activity (GO:0016787), RNA binding (GO:0003723), kinase activity (GO:0016301), lysozyme activity (GO:0003796), chitinase activity (GO:0004568), peroxidase activity (GO:0004601) and hydro-lyase activity (GO:0016836). The 3,4% of the over-represented categories showed no unigene sequences in the reference group.

### *R* genes homologs in *A*. *coronaria*

A tBLASTn-based search of the sequences using as query the full length predicted polypeptide product of 50 *R* genes [36] identified 84 contigs, along with six (1S library) and one (2I library) singletons. The 91 unigenes recognized, related to 16 *R* gene product (those derived from *Vf1*, *Fls2*, *Pbs1*, *Xa21*, *Xa26*, *Rps5*, *Ssi4*, *Rpg1*, *Mlo*, *Hm1*, *Hs1*, *Cf-2*, *Cf-5*, *Cf-9*, *Pto* and *Vrgl1*), distributed between the *R* gene classes NBS-LRR (3), LRR (2), LRR-TM (10), LRR-PK (19), PK (54) and TM (2) with one showing a high level of similarity to *R* genes carrying a Toxin reductase domain (Table 4).

**Table 4:** *Anemone coronaria* unigenes with homology to known Resistance genes.

Unigenes ID	*R* genes	Structure	E-value	Protein ID	Unigenes ID	*R* genes	Structure	E-value	Protein ID
19281	Cf-2	LRR-TM	7,29E-12	AAC15779	8128	Pbs1	PK	5,95E-19	ABR46085
26249	Cf-2	LRR-TM	1,21E-14	AAC15779	18170	Pbs1	PK	2,19E-16	ABR46085
5613	Cf-2	LRR-TM	1,57E-14	AAC15779	9610	Pbs1	PK	8,74E-10	ABR46085
21204	Cf-2	LRR-TM	2,19E-14	AAC15779	15776	Pbs1	PK	9,34E-10	ABR46085
11915	Cf-5	LRR-TM	3,84E-21	AAC78591	15403	Pbs1	PK	1,25E-18	ABR46085
3264	Cf-5	LRR-TM	4,46E-16	AAC78591	24986	Pbs1	PK	2,67E-18	ABR46085
4230	Cf-5	LRR-TM	3,26E-26	AAC78591	5452	Pbs1	PK	3,67E-45	ABR46085
4420	Cf9	LRR-TM	1,70E-17	CAA05274	15392	Pbs1	PK	5,29E-23	ABR46085
21205	Fls2	LRR-PK	1,08E-12	BAB11088	20766	Pbs1	PK	6,85E-15	ABR46085
2470	Fls2	LRR-PK	7,78E-12	BAB11088	15954	Pbs1	PK	3,22E-10	ABR46085
16108	Fls2	LRR-PK	1,35E-10	BAB11088	15959	Pbs1	PK	3,26E-14	ABR46085
191	Fls2	LRR-PK	8,25E-18	BAB11088	917	Pbs1	PK	8,35E-16	ABR46085
9548	Fls2	LRR-PK	1,67E-19	BAB11088	9947	Pbs1	PK	7,38E-26	ABR46085
19550	Fls2	LRR-PK	3,72E-15	BAB11088	GG9DABN02FRDGE	Pbs1	PK	1,79E-14	ABR46085
26189	Hm1	tox reduc	1,03E-10	AAC04333	GG9DABN02HL1AG	Pbs1	PK	2,89E-20	ABR46085
22173	Hs1	LRR-TM	2,84E-19	AAW03319	19538	Pto	PK	1,76E-10	AAB47421
GG9DABN02F9DDG	Hs1	LRR-TM	2,92E-13	AAW03319	7481	Pto	PK	2,38E-13	AAB47421
17299	Mlo	TM	1,55E-13	CAB06083	2969	Pto	PK	6,53E-23	AAB47421
GG9DABN02G8QRM	Mlo	TM	5,53E-12	CAB06083	13334	Pto	PK	4,86E-10	AAB47421
14622	Pbs1	PK	5,85E-14	ABR46085	2466	Pto	PK	4,21E-14	AAB47421
14845	Pbs1	PK	1,44E-23	ABR46085	9388	Pto	PK	1,01E-11	AAB47421
3503	Pbs1	PK	4,16E-10	ABR46085	7553	Pto	PK	4,19E-12	AAB47421
12107	Pbs1	PK	6,30E-13	ABR46085	14902	Pto	PK	8,90E-13	AAB47421
4272	Pbs1	PK	1,37E-21	ABR46085	8930	Pto	PK	1,17E-15	AAB47421
25490	Pbs1	PK	3,40E-15	ABR46085	26150	Pto	PK	3,55E-11	AAB47421
GG9DABN02F3PO5	Pbs1	PK	3,98E-10	ABR46085	18600	Pto	PK	1,21E-12	AAB47421
8545	Pbs1	PK	3,46E-12	ABR46085	GG9DABN02HEM5G	Pto	PK	8,53E-16	AAB47421
19451	Pbs1	PK	1,03E-15	ABR46085	14229	Rps5	NBS-LRR	1,11E-14	AAC26126
16933	Pbs1	PK	1,48E-19	ABR46085	2985	Ssi	NBS-LRR	8,07E-12	AAN86124
17238	Pbs1	PK	1,17E-12	ABR46085	18516	Vf1	LRR	1,89E-14	CAC40825
22527	Pbs1	PK	1,23E-16	ABR46085	19604	Vf1	LRR	6,83E-17	CAC40825
8763	Pbs1	PK	2,31E-25	ABR46085	GG9DABN02HVY07	VRGL1	NBS-LRR	8,78E-12	AAF19148
13820	Pbs1	PK	2,04E-24	ABR46085	919	Xa21	LRR-PK	6,04E-11	AAC80225
10598	Pbs1	PK	1,55E-10	ABR46085	16298	Xa21	LRR-PK	4,41E-10	AAC80225
6752	Pbs1	PK	7,86E-13	ABR46085	4299	Xa21	LRR-PK	8,13E-10	AAC80225
13774	Pbs1	PK	1,21E-12	ABR46085	1845	Xa21	LRR-PK	9,17E-11	AAC80225
14120	Pbs1	PK	5,07E-20	ABR46085	11988	Xa21	LRR-PK	1,40E-21	AAC80225
12619	Pbs1	PK	1,15E-13	ABR46085	18834	Xa21	LRR-PK	1,87E-13	AAC80225
12545	Pbs1	PK	5,78E-12	ABR46085	9452	Xa21	LRR-PK	5,85E-13	AAC80225
13256	Pbs1	PK	5,97E-11	ABR46085	9691	Xa21	LRR-PK	3,10E-10	AAC80225
17036	Pbs1	PK	5,79E-15	ABR46085	8221	Xa26	LRR-PK	6,73E-13	ABK51312
17518	Pbs1	PK	1,34E-19	ABR46085	16817	Xa26	LRR-PK	1,35E-18	ABD36512
22913	Pbs1	PK	1,19E-12	ABR46085	5926	Xa26	LRR-PK	2,17E-13	ABD36512
14431	Pbs1	PK	5,44E-14	ABR46085	11237	Xa26	LRR-PK	3,58E-11	ABK51312
15636	Pbs1	PK	1,84E-11	ABR46085	15836	Xa26	LRR-PK	1,51E-16	ABD36512
8046	Pbs1	PK	5,37E-14	ABR46085					

Unigene products were queried using BLASTx as implemented in the CLC Genomics Workbench 5.0 with the default parameters.

### Identification of differential transcript abundance (DTAs)

In all, 305 DTA genes were identified by comparing transcript abundances between the two libraries. Of these, 253 were present in higher abundance in the 2I library and 52 in lower abundance. In the former set, 234 were not detected in the 1S library and their read number per transcript in the 2I library varied from 15 to 456; similarly 25 of the down-regulated DTAs were not represented in the 2I library, whereas they were present in 15–49 copies in the 1S library (S2 Table). About a half (128) of the DTAs present in the 2I library were of fungal origin, 49 were clearly plant sequences and 7 could have encoded a fungal or a plant protein; the remaining DTAs could not be functionally assigned using BLAST.

### Fungal up-regulated genes

**Fig 3:**
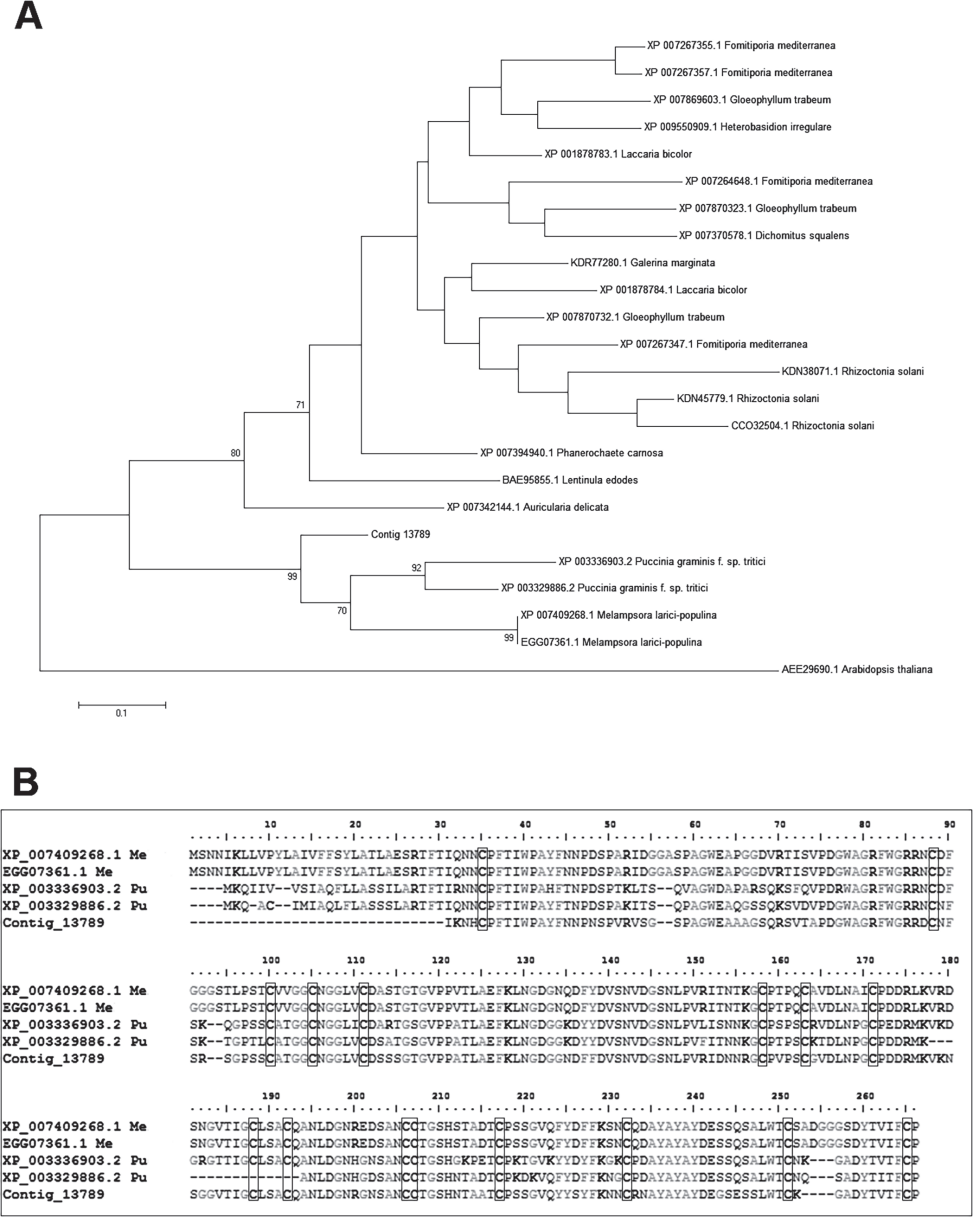
Phylogenetic tree and aligment of thaumatin-like protein. **(A)** *Anemone coronaria* thaumatin-like protein (contig 13789) cluster with *Puccinia graminis* and *Melampsora larici-populina* proteins. TLPs of no-rust fungal taxa cluster in a separate group. Bootstrap values are indicated in relevant nodes. *Arabidopsis thaliana* TLP was used as out-group. (B) Amino acid sequence alignment of five rust TLPs. The conserved 16 cystein residues are highlighted in the boxes.

Among the likely fungal sequences, 118 had homologs in either *P*. *graminis f*. sp. *tritici* or *Melampsora larici-populina* and 78 were associated with a likely function (S2 Table). Of the 30 genes encoding ribosomal proteins (RPs), 26 were likely to have been of fungal origin, reflecting the active protein synthesis exhibited by fungi during the early phase of infection [48]. Genes hydrolytic enzymes acting on plant biopolymers (cellulase), proteinase (subtilase-type proteinase psp3, vacuolar protease A, proteasome subunit 1) and several carbohydrate-active enzymes (glycoside hydrolase, glyceraldehyde-3-phosphate dehydrogenase, enolase, glucose-repressible protein) were well represented, as would be predicted since the invading fungus penetrates the host cells by degrading enzymes [49,50]. Apart from these, a fungal chitinase gene was recognized; this enzyme is used to remodel fungal cell wall during infection, either to promote hyphal invasion and/or to avoid recognition by the host’s defense system [51]. Other strongly represented fungal genes encoded histones, an argonaute-like protein, thiamine synthesis, a mitochondrial thiazole synthesis enzyme and the ubiquitin-conjugating enzyme E2, as was also the case during the infection by rust of both *Populus* sp. and wheat [49]. *P*. *triticina* genes encoding a thiamine synthesis protein and a cyclophilin are also induced *in planta*, as reported by Thara et al. [48]. The first protein is a cofactor controlling the activity of several enzymes involved in the central carbon metabolism [52], whereas cyclophilin is involved in a wide variety of cellular processes, including the response to abiotic stress, the control of cell cycling, the regulation of calcium signaling and control of transcriptional repression [53–55]. The transcription of a gene encoding a thaumatin-like protein (TLP) may reflect the fungus’ attempt to interfere with the host’s defense signaling apparatus [56,57]; the presence of this protein has been noted in the *Melampsora* secretome [58,59]. The phylogenetic tree of TLP resolved the entries into two major branches. One includes rust fungal proteins only (*P*. *graminis* and *M*. *larici-populina* and *T*. *discolor*), the other groups proteins of all other fungal taxa. *T*. *discolor* TLP is well separated from *P*. *graminis* and *M*. *larici-populina* proteins with a bootstrap of 99% (Fig. 3A). Amino acid sequence of *T*. *discolor* TLP shares with rust TPLs the 16 conserved cysteine residues that characterize the large type TLPs [60].

### Plant up-regulated genes

Among the *A*. *coronaria* genes up-regulated in the 2I library, 17 had homologs in *V*. *vinifera*, five in *A*. *thaliana* and the remainder in another species. The BLAST assignment of these DTAs is given in S2 Table. In what follows, the function of plant DTAs with potential relevance for host/pathogen interaction is explored.

**Ribosomal proteins (RPs) genes.** Four genes encoding 40S or 60S RPs were up-regulated in the infected plants, suggesting not only an increased level of protein synthesis induced by the infection process, but also the promotion of a suite of extra ribosomal activities, such as DNA repair, apoptosis, inflammation, tumorigenesis and transcriptional regulation [61]. The ribosomal protein S3 (RPS3), which is a component of the eukaryotic 40S ribosome is known to be involved in certain host–pathogen interactions [62].

**Fig 4:**
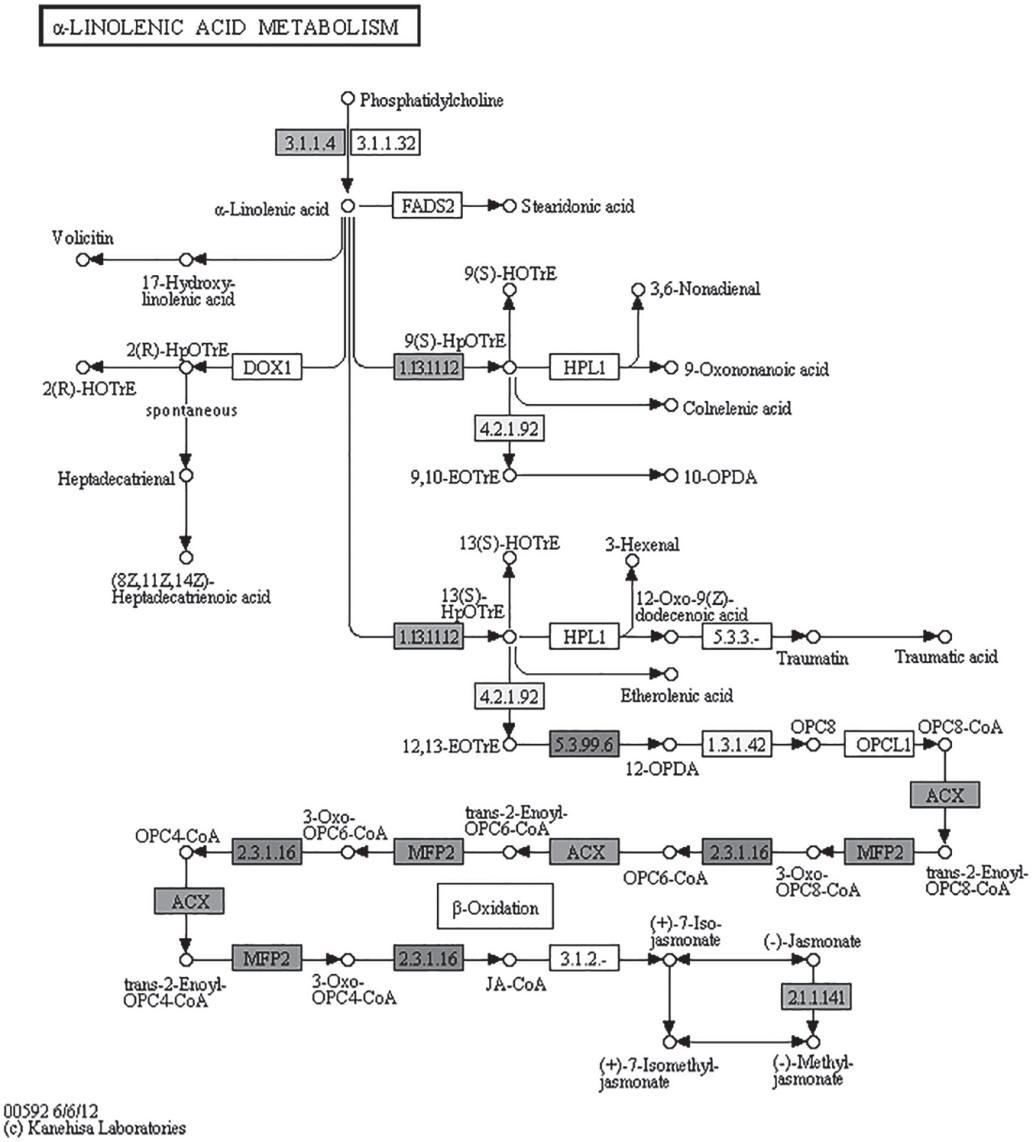
KEGG pathway of α-linoleic acid metabolism. *Anemone coronaria* transcrips involved in jasmonic acid metablolic pathway are highlighted by grey tone. 12-oxophytodienoate reductase 2 (EC: 5.3.99.6) and acyl-CoA oxidase (ACX) are upregulated during *Tranzschelia discolor* infection.

**Genes involved jasmonate (JA) signaling.** Among the genes up-regulated in the infected plants were six which encode various proteins involved in JA signaling; these included those encoding 12-oxophytodienoate reductase 2 [63] and acyl-CoA oxidase [64], which are both components of JA synthesis, two JA-induced proteins, one JA signaling repressor (TIFY 3B, also known as JAZ12) and strictosidine synthase 1 [65]. JAZ proteins were degraded on perception of jasmonyl-isoleucine (JA-Ile, active form of JA) allowing the JA-Ile dependent gene expression [66,67]. Strictosidine synthase 1, a key enzyme in alkaloid biosynthesis, was induced by plant defence signalling compounds, such as salicylic acid (SA), ethylene and methyl jasmonate [65]. The Kegg analysis of the jasmonate biosynthetic pathway proves that six genes, in addition to the two up regulated, were identified by the trascriptome sequencing (Fig. 4).

***R* gene.** Among *R* genes identified by tBLASTn analysis, only polygalacturonase inhibitor-like (PGIPs), which harbor a leucine rich repeat and a transmembrane domain (LRR-TM) was significantly up-regulated. PGIPs can inhibit fungal endopolygalacturonases (PGs) which are responsible for breaking down the host cell wall and their encoding genes are typically induced by pathogen infection [68]. The levels of PGIP were correlated with an increased resistance to fungi in raspberry fruits [69], in older bean hypocotyls [70] and in tomato transgenic plants [71].

**Fig 5:**
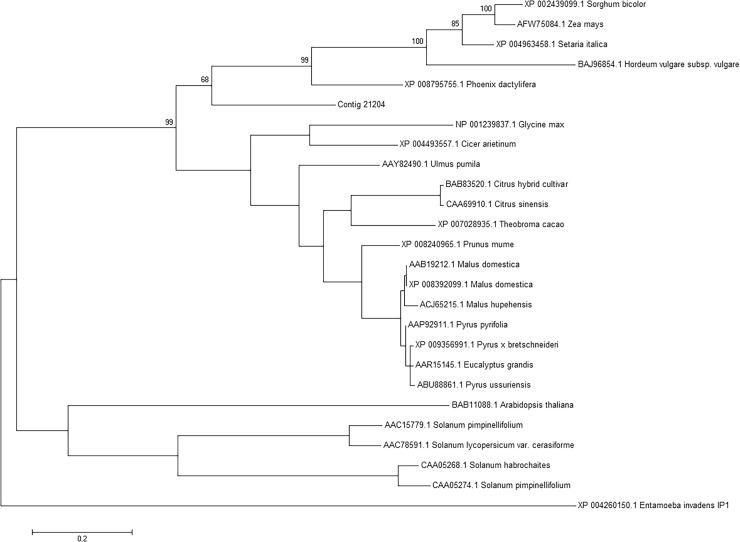
Phylogenetic tree of PGIP. *Anemone coronaria* PGIP (contig 21204) clusters with those of Monocotyledon species. Dicotyledon PGIPs cluster in two separate groups. Bootstrap values are indicated in relevant nodes. *Entamoeba invadens* PGIP protein was used as out-group.

*A*. *coronaria* PGIP clusters together with *Monocotyledon*. This unexpected result draws a parallel with the phylogenetic classification of the species into the early diverging *Eudicotyledon* clade [72]. PGIP of core *Eudicotyledon* clusters into two well separated subgroups (Fig. 5).

**Genes encoding pathogenesis related protein (PR).** Ten *PR* genes were up regulated in the infected plants; they encoded either a chitinase (three genes), a bacterial-induced peroxidases (two genes), a defensin-like protein 13, a major latex protein (MLP28), an S-norcoclaurine synthase-like (NCS) enzyme, a thionin and a metallothionein. Chitinases are an important group of PR proteins because chitin is the major component of many fungal cell walls [73,74]. Duplessis et al. [75] have shown that the early expression of chitinase is needed for an incompatible *Populus*-*Melampsora* interaction.

**Fig 6:**
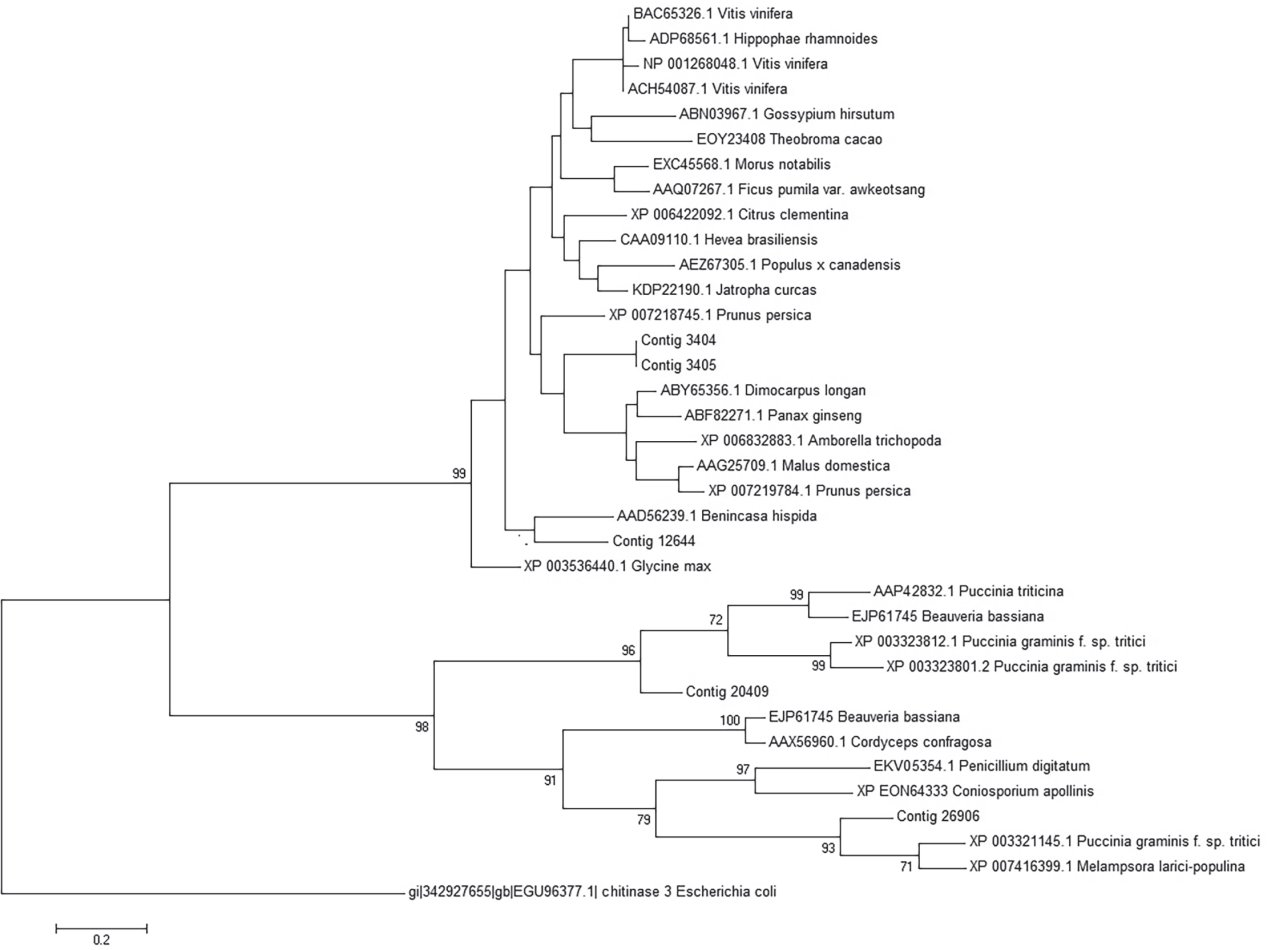
Phylogenetic tree of chitinases. Analysis resolves family 18 chitinases into two main branches: the first includes plant chitinases and the second bring together fungal chitinases. *Escherichia coli* chitinase was used as out-group.

Phylogenetic analysis resolved fungal and plant family 18 chitinases [76] into two main branches. The first branch contains plant chitinases, comprehensive of *A*. *coronaria* contigs 3404, 3405 and 12644. The second branch bring together fungal chitinases and include *T*. *discolor* contigs 26906 and 20409, that is strongly over expressed during plant infection (Fig. 6).

The large family of peroxidase represent enzymes [77] which contribute to plant disease resistance in several ways: they act to strengthen the host cell wall via deposition of lignin, which acts as a physical barrier against pathogen ingress [78] and also produce toxic radicals such as hydrogen peroxides [79,80]. The defensin-like protein 13 (PDF1.1) belong to a family of antimicrobial peptides which are intimately involved in determining innate immunity [81,82]. MLP28 and NCS were homologous to PR10 proteins, that are thought to participate in the defense of plants against microorganisms and fungi [83]. The MLP protein family has been associated with pathogen defense, although how they act remains unknown. NCS catalyzes the first committed step in the synthesis of benzylisoquinoline alkaloids [84]. Thionin (PR 13) is a well studied compound known to be able to permeate pathogen membranes [85]; the presence of these compounds is frequently induced in the leaf and they are present at high levels in floral tissue [86]. Finally, the metallothioneins are small cystein-rich proteins involved in correcting for imbalances in metal ions and the regulation of homeostasis under various stresses. Their participation in plant defense is thought to involve the induction of reactive oxygen species (ROS) and the suppression of ROS scavenging enzymes [87–89]. Nishimura et al. [90] have recently proposed that, they could also be used by the plant to control the synthesis of pathogen toxins via inhibition of zinc absorption by the pathogen.

**Other up-regulated genes putatively involved in defense response.** Nine other genes associated with the defense response were up-regulated in the infected plants. These encoded caffeic acid 3-O-methyltransferase (COMT), Cytochrome P450 (CYP 450), Early responsive to dehydration (ERD), Flavonol synthase (FLS), Heat shock proteins (HSPs), Lipid binding protein (LTPs), SNARE-interacting protein KEULE (KEU) and UDP-glucose transglucosylase-like protein. Tremblay et al. [91] used the up-regulation of COMT genes (their product is an important component of phenylpropanoid synthesis) as a marker for the activation of the plant defense response. CYP 450 contributes to oxidative metabolism and the production of ROS and is reportedly involved in the hypersensitive response (HR) to pathogen infection [92]. Some CYPs participate in the synthesis of the defense-associated compounds: lignin, phytoalexins and anthocyanins [93]. The ERD gene family comprises at least 21 members in Arabidopsis and have been identified as part of the immediate response to drought stress. Altering the level of ERD15 transcript not only had an effect on the plant’s abiotic stress tolerance but also on its level of disease resistance [94]. FLS converts both flavanones and dihydroflavonols to their related flavonols; the enzyme is a bifunctional dioxygenase, with certain hydroxylation and desaturation activities [95,96]. While many studies have indicated a role for flavonoids in disease resistance, the multi-functionality of these compound complicates the interpretation of results. [97]. Two *HSP* genes were up-regulated: *HSP90* product has a role in signal transduction during the plant defenses response [98], while HSP23.6 accumulates during the systemic infection of *Actinidia chinensis* with *P*. *syringae pv*. *Actinidiae* [99]. Silencing of *HSP90* in *N*. *benthamiana* compromises not only induction of the HR, but also non host resistance [100]. HSP90, in conjunction with other proteins, is also known to modulate N gene-mediated resistance to *Tobacco mosaic virus* in tobacco and RPS2 and RPM1-mediated resistance to *P*. *syringae* in Arabidopsis [101,102]. LTPs are able to transfer phospholipids between membranes and to bind fatty acids *in vitro* and are putatively involved in cutin synthesis, surface wax formation, defenses against pathogen and adaptation to environmental changes [103]. KEU interacts with the SNARE domain present in certain genes active in plant defense [104]. Loss-of-function of gene encoding SNARE enable elevated levels of host cell entry either by non adapted fungal species and delay in the formation of localized cell wall appositions [105]. UDP-glucose transglucosylase is thought to be involved in the synthesis of cell wall polysaccharide [106] and is active in grapevine plants exposed to pathogen infection [107]. A total of 16 other genes with no known involvement in pathogen defense were also represented by enhanced transcription the 2I library (S2 Table).

### Down-regulated genes

Among the down-regulated genes, 32 were of plant origin, one of viral origin and one shared homology with both bacterial and plant proteins. The remaining 18 genes gave no BLAST hit. The genes had homologs in *A*. *thaliana* (nine), *V*. *vinifera* (eight) and *R*. *comunis* (four), with the other nine related to genes from *G*. *max*, *P*. *tricocarpa*, *M*. *truncatula* (S2 Table). In what follows, the function of plant DTAs with potential relevance for host/pathogen interaction is explored.

**Cell wall associated genes.** Six genes encoding components of the constitutive defense response were down-regulated in the infected plants. Two involved cell wall-associated hydrolases which act to degrade and reorganize the cell wall [108,109]; one was a cellulose synthase-like protein (CesA superfamily) which synthesizes cellulose and is required for secondary cell wall formation, one was a xyloglucan endotransglucosylase (XTH), one was a white-brown-complex (WBC) ATP-binding cassette (ABC) transporters family and one was a protein containing a galactose-binding domain-like fold (lectins). Affecting the cell wall’s integrity by inhibiting cellulose synthesis induces the activation of a number of host defense mechanism designed to produce an environment enriched with respect to antimicrobial compounds [110]. XTH restructures and loosens the xyloglucan network in the cell wall, thereby enabling cell expansion [111]. In *A*. *thaliana*, Gruner et al. [112] have shown that several genes encoding XTH, arabinogalactans, expansin- and extension-like proteins and polygalacturonase are all strongly down-regulated during the development of the systemic acquired resistance (SAR) process. Certain ABC transporters are known to be important for assuring the movement of cutin monomers [113] and others in the resistance to a number of fungal pathogens in wheat [114]. Together with other defense genes work in a sequential and concerted manner to result in a hypersensitive response to *Puccinia striiformis* infection [115]. Lectins, which contain a galactose-binding domain-like fold, act to bind specific ligands (such as, for example, cell surface-attached carbohydrate) and represent the only plant proteins capable of recognizing and binding the glycol-conjugates present on the outer surface of bacteria and fungi [116]. While the down regulation of CesA superfamily and XTH may activate certain defense pathways, the suppression of genes encoding cell wall associated hydrolases, ABC transporters and lectins is quite conceivably one of the means whereby *T*. *discolor* overcomes the host’s constitutive defense machinery.

**Fig 7:**
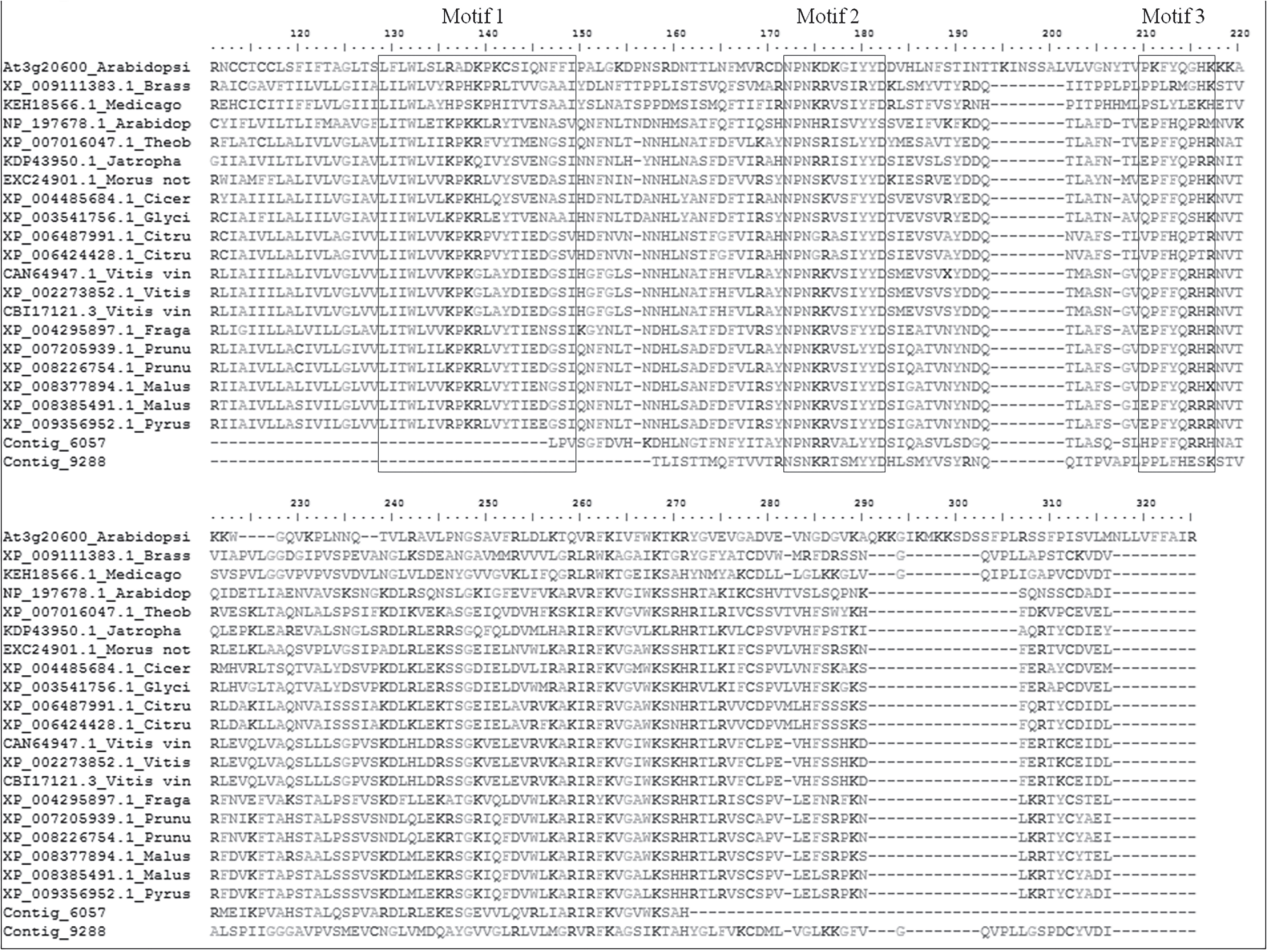
Aligment of NDR1 proteins. *Anemone coronaria* contigs 6057 and 9288 are aligned with selected member of NDR1 proteins. Motif 1, motif 2 and 3 of NDR1/HIN-like (NHL) protein superfamily are highlighted in the boxes.

Two genes encoding a product with significant homology to NON-RACE-SPECIFIC DISEASE RESISTANCE1 (NDR1), a plasma membrane-localized protein were down regulated. NDR1 is involved in the maintenance of the integrity of the cell wall/plasma membrane connection and represents a key signaling component during pathogen infection [117]. In Arabidopsis a member of the CC-NBS-LRR R protein require the signaling gene NDR1 for full activity [118]. Alignment of *A*. *coronaria* NDR1 deduced protein with the NCBI nr database proteins identifies motif 2 and 3 of the three NDR1/ HIN1-like (NHL) protein superfamily [119,120]. Motif 1 was not covered by the *A*. *coronaria* sequence (Fig. 7). Phylogenetic analysis together with down regulation during *T*. *discolor* infection provide evidence that *A*. *coronaria* NDR1 genes are credible candidate of the fungus to establish biotrophic relationship.

**Other down-regulated genes putatively involved in defense response.** Five genes encoding defense-associated proteins were down-regulated in the infected plants: one was a *myb* transcription factor and the others encoded a presqualene diphosphate phosphatase (PSDPase), a UDP-glycosyltransferase 74E2-like protein (UGT), a peptide transporter with homology to PTR3 and a secologanin synthase-like protein. The large family of *myb* factors includes many involved in regulating the defense response [121]. PdMYB3, for example, is more strongly activated in disease susceptible than in disease resistant *Prunus domestica* cultivars [122]. Fukunaga et al. [123] have shown that a human PSDPase (which converts PSDP to a monophosphate form) is important for maintaining cell function in the face of disease pressure. In the *A*. *coronaria* / *T*. *discolor* interaction, the down-regulation of the gene encoding PSDPase would likely shift the PSDP pathway in the direction of oxidosqualene, the precursor of membrane sterols, brassinosteroids, saponins and other defense compounds [124]. Most pathogen-induced SA is glycosylated by UGT to form the non-toxic SA 2-O-β-D-glucoside. The combination of SA methylation, amino acid conjugation and glycosylation forms an intimate part of the plant defense response [125,126]. A UGT loss-of-function mutant has been shown to express an enhanced level of SAR [127]. PTR3 is regulated by both SA and JA. Its *A*. *thaliana* homolog AtPTR3 is induced by the presence of the *P*. *syringae* pathogen [128], while loss-of-function mutants show accentuated susceptibility to both *Erwinia carotovora* and *P*. *syringae*. Secologanin synthase catalyzes the oxidative cleavage of loganin into secologanin [129], a component of the terpenoid indole alkaloids proposed to be involved in plant defense [130]. The down-regulation of *myb*, PSDPase and UGT is suggestive of the activation of host defense against *T*. *discolor*, while the down-regulation of PTR3 and the gene encoding the secologanin synthase-like protein may reflect pathogen growth and the establishment of a compatible host/pathogen interaction.

### Validation of DTA by real-time quantitative PCR (qPCR)

**Fig 8:**
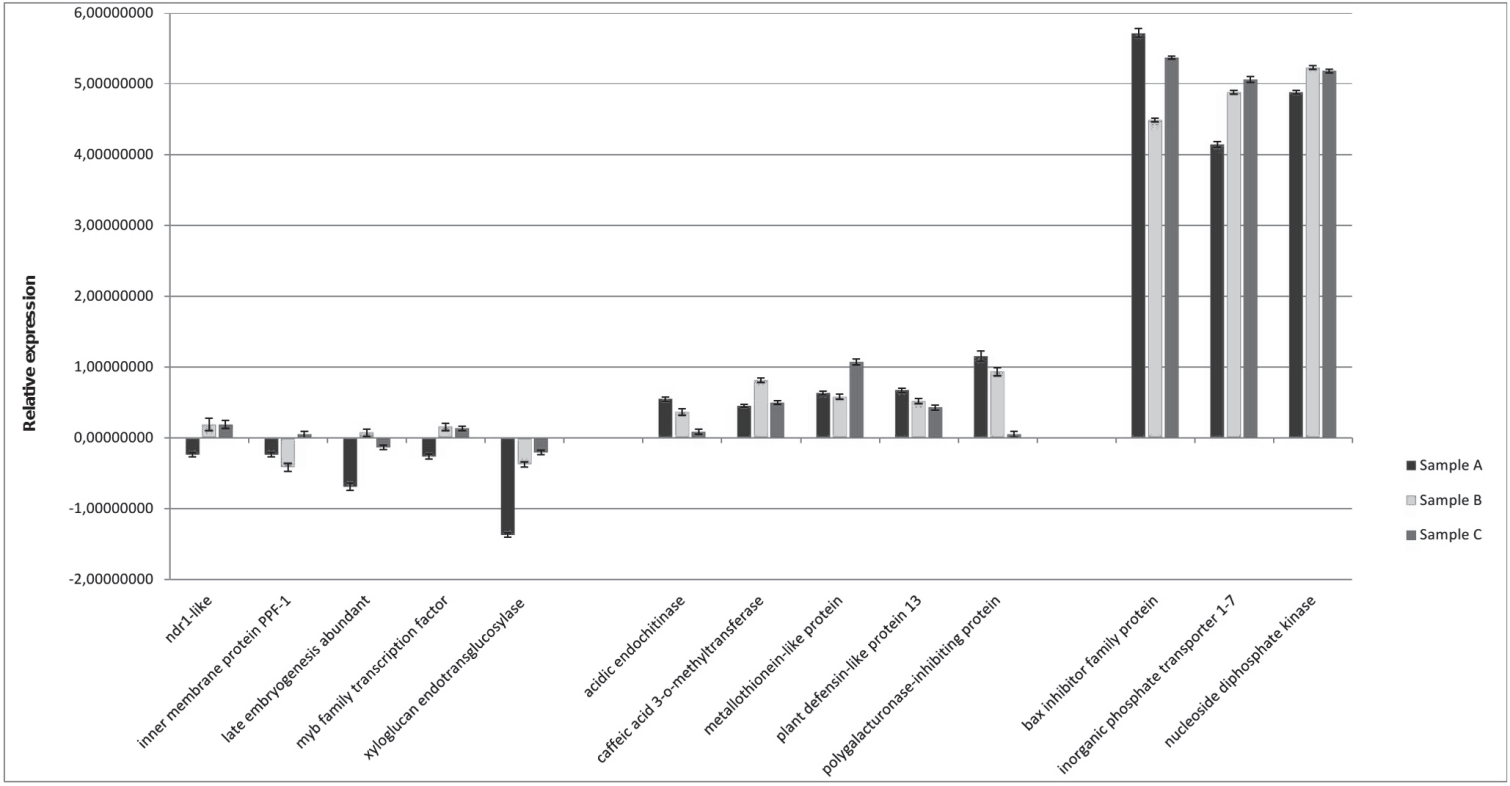
Gene expression in *Anemone coronaria* infected with *Tranzschelia discolor*. Expression analysis was conducted among sample A (plants analyzed by pyrosequencing) and samples B and C, each composed of five distinct uninfected and infected plants. Ten plant DTAs genes (five up- and five down-regulated) putatively involved in the response to *Tranzschelia discolor* infection and three fungal genes, were tested. The data were normalized using *Anemone coronaria* 18s rRNA gene as the reference. Expression analysis was performed in triplicate on three biological replicates. Transcript abundance data were expressed in the form mean ± standard error (SE).

The qPCR analysis based on ten plant and three fungal target results (unigenes) confirmed that the 3’ sequencing of non-normalized libraries was informative with respect to recognizing DTAs. qPCR data of sample A (plants analyzed by pyrosequencing) were compared with qPCR data of samples B and C, each composed of five distinct uninfected and infected plants. Significant differences in expression was observed for the 13 genes tested (Fig. 8 and Table 1). The up regulated genes showed the same behavior, whereas down regulated genes varied significantly likely as a result of sample bias at low expression levels. In addition, divergences in level of gene expression may reflect time points of the plant/pathogen interaction or the genetic eterogenicity in *A*. *coronaria* population.

## Conclusions

Until now, the amount of genomic information for *A*. *coronaria* in the public domain has been limited to one EST and 12 DNA sequences. This has now been rectified by the acquisition of 600,000 cDNA sequences, assembled into over 27,000 contigs. The estimated coverage of the gene content of the species was 71%, with almost all genes being represented by at least one read. ESTcalc and UCO analysis also estimated that almost all gene were represented by at least one read. Taken together these data demonstrate the potential of 3’ sequencing, although an half 454 plate only was sequenced.

Biotrophic fungi require the presence of living host tissue for their survival. Rusts such as *P*. *graminis*, *Melampsora* spp. and *T*. *discolor* are obligate biotrophs which often require two phylogenetically non-related hosts [49]. They have evolved specialized structures, haustoria, formed within host tissue to efficiently acquire nutrients and suppress host defense responses [131].

In cultivated *A*. *coronaria* susceptible plants, a compatible interaction occurs when seedling challenge *T*. *discolor* teliospores formed on *Prunus* leaf. In this phase the pathogen overcome constitutive defenses including many preformed barriers such as cell walls and waxy epidermal cuticle The *A*. *coronaria* transcriptome included the products of 16 of the 50 *R genes* described in *A*. *thaliana* [36], but in infected *A*. *coronaria* leaf tissue, only one of them was up-regulated. In the meanwhile two NDR1 genes involved in activation of CC-NBS-LRR *R genes* were down regulated. As previously reported for several plant / rust interaction [6], the ability of *T*. *discolor* effectors to escape *A*. *coronaria* R protein recognition and activation is likely the key of compatibility. During leaf colonization by fungal hyphae, field grown *A*. *coronaria* plants, activate their own immune systems and overexpress PR proteins as chitinases, involved in degradation of fungal cell wall chitin, peroxidases that may have a role in inhibiting the hyphal extention, and several additional protein (defensin-like, metallothionein, MLP-like protein 28-like, S-norcoclaurine synthase-like, thionin precursor).

The response of the plant to fungal invasion includes in addition the up-regulation of several genes associated with cellular defense. Some of these, encoding peroxidase, CYP 450, superoxide dismutase Cu/Zn chloroplast and metallothionein are involved in HR that lead to cell death and stops biotrophism. SAR is an important component of the defensive armoury of plants; it provides protection against infection by a broad range of pathogens [11,13–15,132]. The phenomenon is co-ordinated by various phytohormones. Several genes involved in JA signaling were induced in the infected *A*. *coronaria* plants, which suggests the activation of SAR, as does the induction of the genes *ERD*, *PDF1*.*1*, *ABA glucosyl transferase* and the down-regulation of *XTH* and *UGT*. Taken together these data show that either constitutive or *R gene* mediated defense are overcome in *A*. *coronaria* by *T*. *discolor*. *A*. *coronaria* activate both primary and secondary immune system that trigger HR. SAR is induced simultaneously. Despite plant reaction *T*. *discolor* strongly affect *A*. *coronaria* gene expression to support mating, sporulation and completing its life cycle. Transcriptome sequencing is a convenient choice to investigate a complex traits as plant pathogen interaction despite the wide genome size of *A*. *coronaria* (165.28 MB corresponding to 137X *A*. *thaliana*). To fulfill an exhaustive set of knowledge, transcriptome data provided in this work need to be implemented with sequencing of full coding and regulatory regions together with an analysis of RNA interference. Next-generation re sequencing of selected genomic regions of a large amount of accession represents a powerful approach to identify the complete spectrum of DNA sequence variants [133]. This technology is a powerful approach to discover resistance alleles in candidate genes selected among the DTA s during *A*. *coronaria* / *T*. *discolor* interaction. A short cut strategy to bread resistant genotype was proposed after the advent of genetic engineering [134]. Coding sequences of the differentially over expressed *A*. *coronaria* gene can be expressed at early stage of pathogen infection by constitutive, inducible or tissue specific promoters to effectively counteract the disease. On the other side, silencing of genes that are activated directly by pathogen effectors or indirectly by the guardee proteins may result in an attenuated virulence. Targeted mutagenesis is the most recent tool to disrupt *Avr* gene targets [135] and to confer new recognition specificities [136]. The major constrain for utilization of genetic engineering and targeted mutagenesis in *A*. *coronaria*, is the lack of a reliable transformation method and transient system for expression of nucleases respectively.

The *Ranunculaceae* family belongs to an ancient eudicotyledonous clade [72] which includes a number of both ornamental and medicinal species. The present study represents the first analysis of the transcriptome of such an early diverging species [137]. The identification of gene sequences of the pathogen *T*. *discolor* will enable its interaction with its primary host (*Prunus* spp.) to be investigated: the latter genus of trees and shrubs is much utilized both for its fruit and flowers. The DTA genes identified here should provide a basis for understanding the *A*. *coronaria / T*. *discolor* interaction and leads for biotechnology-based disease resistance breeding.

## Supporting Information

S1 Table GO categories differentially represented between the 2I (test set) and 1S (references set) libraries. (XLS)

S2 Table Genes differentially expressed on the base of transcript abundance between the 2I and 1S libraries. Sheet one: up regulated genes of *Tranzschelia discolor*; sheets two and three: up and down regulated genes of *Anemone coronaria* respectively.
(XLSX)

S1 Fig Size distribution of the 454 raw reads. 2I represents infected library and 1S represent uninfected library.
(TIF)

S2 Fig Size distribution of the contigs. The contigs were mass assembled from the two libraries; the mean length is 377 nt.
(TIF)

S3 Fig Size distribution of sequences with or without BLASTx. The 50.5% of the predicted translation products shared significant homology with known protein sequences deposited in GenBank and 1.7% with hypothetical proteins, leaving 47.8% of the sequences unannotated. The proportion of sequences lacking any BLASTx alignment and shorter than 250 nt was 42.1%.
(TIF)

S4 Fig Top-hit species distribution. *Vitis vinifera* (grape) is the most frequently occurring species, followed by *Populus tricocarpa* (black cottonwood), *Ricinus communis* (the castor oil plant), *Glycine max* (soybean) and *Puccinia graminis* (cereal stem black rust).
(TIF)

## References

[1] Nissim Y, Jinggui F, Arik S, Neta P, Uri L, Avner C (2004) Phenotypic and genotypic analysis of a commercial cultivar and wild populations of Anemone coronaria. Euphytica 136: 51–62.

[2] Lopez-Franco RM, Hennen JF (1990) The Genus Tranzschelia (Uredinales) in the Americas. Systematic Botany 15: 560–591.

[3] Szabo LJ, Bushnell WR (2001) Hidden robbers: The role of fungal haustoria in parasitism of plants. Proceedings of the National Academy of Sciences 98: 7654–7655. 11438718 10.1073/pnas.151262398 PMC35395

[4] Hok S, Attard A, Keller H (2010) Getting the most from the host: how pathogens force plants to cooperate in disease. Mol Plant Microbe Interact 23: 1253–1259. 10.1094/MPMI-04-10-0103 20636104

[5] Dodds PN, Rathjen JP (2010) Plant immunity: towards an integrated view of plant-pathogen interactions. Nat Rev Genet 11: 539–548. 10.1038/nrg2812 20585331

[6] Schneider DJ, Collmer A (2010) Studying plant-pathogen interactions in the genomics era: beyond molecular Koch's postulates to systems biology. Annu Rev Phytopathol 48: 457–479. 10.1146/annurev-phyto-073009-114411 20687834

[7] Glazebrook J (1999) Genes controlling expression of defense responses in Arabidopsis. Curr Opin Plant Biol 2: 280–286. 10458996 10.1016/S1369-5266(99)80050-8

[8] de Wit PJ (2007) How plants recognize pathogens and defend themselves. Cell Mol Life Sci 64: 2726–2732. 17876517 10.1007/s00018-007-7284-7 PMC11136299

[9] Jones JDG, Dangl JL (2006) The plant immune system. Nature 444: 323–329. 17108957 10.1038/nature05286

[10] Vivier E, Malissen B (2004) Innate and adaptive immunity: specificities and signaling hierarchies revisited. Nature immunology 6: 17–21. 10.1038/ni1153 PMC7097365 15611777

[11] Van Loon L (1997) Induced resistance in plants and the role of pathogenesis-related proteins. European Journal of Plant Pathology 103: 753–765.

[12] Grant SR, Fisher EJ, Chang JH, Mole BM, Dangl JL (2006) Subterfuge and manipulation: type III effector proteins of phytopathogenic bacteria. Annu Rev Microbiol 60: 425–449. 16753033 10.1146/annurev.micro.60.080805.142251

[13] Fritig B, Heitz T, Legrand M (1998) Antimicrobial proteins in induced plant defense. Curr Opin Immunol 10: 16–22. 9523105 10.1016/s0952-7915(98)80025-3

[14] Kohler A, Schwindling S, Conrath U (2002) Benzothiadiazole-Induced Priming for Potentiated Responses to Pathogen Infection, Wounding, and Infiltration of Water into Leaves Requires the NPR1/NIM1 Gene in Arabidopsis. Plant Physiology 128: 1046–1056. 11891259 10.1104/pp.010744 PMC152216

[15] Jung HW, Tschaplinski TJ, Wang L, Glazebrook J, Greenberg JT (2009) Priming in systemic plant immunity. Science 324: 89–91. 10.1126/science.1170025 19342588

[16] Mardis ER (2008) Next-generation DNA sequencing methods. Annu Rev Genomics Hum Genet 9: 387–402. 10.1146/annurev.genom.9.081307.164359 18576944

[17] Mazumder B, Seshadri V, Fox PL (2003) Translational control by the 3′-UTR: the ends specify the means. Trends in Biochemical Sciences 28: 91–98. 12575997 10.1016/S0968-0004(03)00002-1

[18] Morozova O, Hirst M, Marra MA (2009) Applications of new sequencing technologies for transcriptome analysis. Annu Rev Genomics Hum Genet 10: 135–151. 10.1146/annurev-genom-082908-145957 19715439

[19] Marguerat S, Bahler J (2010) RNA-seq: from technology to biology. Cell Mol Life Sci 67: 569–579. 10.1007/s00018-009-0180-6 19859660 PMC2809939

[20] Bloom JS, Khan Z, Kruglyak L, Singh M, Caudy AA (2009) Measuring differential gene expression by short read sequencing: quantitative comparison to 2-channel gene expression microarrays. BMC Genomics 10: 221 10.1186/1471-2164-10-221 19435513 PMC2686739

[21] Marioni JC, Mason CE, Mane SM, Stephens M, Gilad Y (2008) RNA-seq: an assessment of technical reproducibility and comparison with gene expression arrays. Genome Res 18: 1509–1517. 10.1101/gr.079558.108 18550803 PMC2527709

[22] t Hoen PA, Ariyurek Y, Thygesen HH, Vreugdenhil E, Vossen RH, de Menezes RX, et al (2008) Deep sequencing-based expression analysis shows major advances in robustness, resolution and inter-lab portability over five microarray platforms. Nucleic Acids Res 36: e141 10.1093/nar/gkn705 18927111 PMC2588528

[23] Eveland AL, McCarty DR, Koch KE (2008) Transcript profiling by 3'-untranslated region sequencing resolves expression of gene families. Plant Physiol 146: 32–44. 18024554 10.1104/pp.107.108597 PMC2230554

[24] Nagalakshmi U, Wang Z, Waern K, Shou C, Raha D, Gerstein M, et al (2008) The transcriptional landscape of the yeast genome defined by RNA sequencing. Science 320: 1344–1349. 10.1126/science.1158441 18451266 PMC2951732

[25] Sultan M, Schulz MH, Richard H, Magen A, Klingenhoff A, Scherf M, et al (2008) A global view of gene activity and alternative splicing by deep sequencing of the human transcriptome. Science 321: 956–960. 10.1126/science.1160342 18599741

[26] Alagna F, D'Agostino N, Torchia L, Servili M, Rao R, Pietrella M, et al (2009) Comparative 454 pyrosequencing of transcripts from two olive genotypes during fruit development. BMC Genomics 10: 399 10.1186/1471-2164-10-399 19709400 PMC2748093

[27] Barakat A, Staton M, Cheng CH, Park J, Yassin NB, Ficklin S, et al (2012) Chestnut resistance to the blight disease: insights from transcriptome analysis. BMC Plant Biol 12: 38 10.1186/1471-2229-12-38 22429310 PMC3376029

[28] Wang W, Wang Y, Zhang Q, Qi Y, Guo D (2009) Global characterization of Artemisia annua glandular trichome transcriptome using 454 pyrosequencing. BMC Genomics 10: 465 10.1186/1471-2164-10-465 19818120 PMC2763888

[29] Sun C, Li Y, Wu Q, Luo H, Sun Y, Song J, et al (2010) De novo sequencing and analysis of the American ginseng root transcriptome using a GS FLX Titanium platform to discover putative genes involved in ginsenoside biosynthesis. BMC Genomics 11: 262 10.1186/1471-2164-11-262 20416102 PMC2873478

[30] Rowland LJ, Alkharouf N, Darwish O, Ogden EL, Polashock JJ, Bassil NV, et al (2012) Generation and analysis of blueberry transcriptome sequences from leaves, developing fruit, and flower buds from cold acclimation through deacclimation. BMC Plant Biol 12: 46 10.1186/1471-2229-12-46 22471859 PMC3378433

[31] Der JP, Barker MS, Wickett NJ, dePamphilis CW, Wolf PG (2011) De novo characterization of the gametophyte transcriptome in bracken fern, Pteridium aquilinum. BMC Genomics 12: 99 10.1186/1471-2164-12-99 21303537 PMC3042945

[32] Wang Y, Zeng X, Iyer NJ, Bryant DW, Mockler TC, Mahalingam R (2012) Exploring the switchgrass transcriptome using second-generation sequencing technology. PLoS One 7: e34225 10.1371/journal.pone.0034225 22479570 PMC3315583

[33] Götz S, García-Gómez JM, Terol J, Williams TD, Nagaraj SH, Nueda MJ, et al (2008) High-throughput functional annotation and data mining with the Blast2GO suite. Nucleic acids research 36: 3420–3435. 10.1093/nar/gkn176 18445632 PMC2425479

[34] Benjamini Y, Hochberg Y (1995) Controlling the false discovery rate: a practical and powerful approach to multiple testing. Journal of the Royal Statistical Society Series B (Methodological): 289–300.

[35] Blüthgen N, Brand K, Čajavec B, Swat M, Herzel H, Beule D (2004) Biological profiling of gene groups utilizing Gene Ontology. arXiv preprint q-bio/0407034.

[36] Liu Z, Crampton M, Todd A, Kalavacharla V (2012) Identification of expressed resistance gene-like sequences by data mining in 454-derived transcriptomic sequences of common bean (Phaseolus vulgaris L.). BMC Plant Biol 12: 42 10.1186/1471-2229-12-42 22443214 PMC3353201

[37] Wall PK, Leebens-Mack J, Chanderbali AS, Barakat A, Wolcott E, Liang H, et al (2009) Comparison of next generation sequencing technologies for transcriptome characterization. BMC Genomics 10: 347 10.1186/1471-2164-10-347 19646272 PMC2907694

[38] Anders S, Huber W (2010) Differential expression analysis for sequence count data. Genome biol 11: R106 10.1186/gb-2010-11-10-r106 20979621 PMC3218662

[39] Kimbrel JA, Di Y, Cumbie JS, Chang JH (2011) RNA-Seq for Plant Pathogenic Bacteria. Genes 2: 689–705. 10.3390/genes2040689 24710287 PMC3927590

[40] Cantu D, Govindarajulu M, Kozik A, Wang M, Chen X, Kojima KK, et al (2011) Next Generation Sequencing Provides Rapid Access to the Genome of *Puccinia striiformis* f. sp. *tritici*, the Causal Agent of Wheat Stripe Rust. PLoS ONE 6: e24230 10.1371/journal.pone.0024230 21909385 PMC3164196

[41] Tamura K, Peterson D, Peterson N, Stecher G, Nei M, Kumar S (2011) MEGA5: Molecular Evolutionary Genetics Analysis Using Maximum Likelihood, Evolutionary Distance, and Maximum Parsimony Methods. Molecular Biology and Evolution 28: 2731–2739. 10.1093/molbev/msr121 21546353 PMC3203626

[42] Laura M, Borghi C, Regis C, Cassetti A, Allavena A (2013) Ectopic expression of Kxhkn5 in the viviparous species Kalanchoe× Houghtonii induces a novel pattern of epiphyll development. Transgenic research 22: 59–74. 10.1007/s11248-012-9628-9 22829336

[43] Livak KJ, Schmittgen TD (2001) Analysis of relative gene expression data using real-time quantitative PCR and the 2(-Delta Delta C(T)) Method. Methods 25: 402–408. 11846609 10.1006/meth.2001.1262

[44] Mann HB, Whitney DR (1947) On a test of whether one of two random variables is stochastically larger than the other. The annals of mathematical statistics: 50–60.

[45] Logacheva MD, Kasianov AS, Vinogradov DV, Samigullin TH, Gelfand MS, Makeev VJ, et al (2011) De novo sequencing and characterization of floral transcriptome in two species of buckwheat (Fagopyrum). BMC Genomics 12: 30 10.1186/1471-2164-12-30 21232141 PMC3027159

[46] Parchman TL, Geist KS, Grahnen JA, Benkman CW, Buerkle CA (2010) Transcriptome sequencing in an ecologically important tree species: assembly, annotation, and marker discovery. BMC Genomics 11: 180 10.1186/1471-2164-11-180 20233449 PMC2851599

[47] The EUAGP, Bevan M, Bancroft I, Bent E, Love K, Goodman H, et al (1998) Analysis of 1.9[thinsp]Mb of contiguous sequence from chromosome 4 of Arabidopsis thaliana. Nature 391: 485–488. 9461215 10.1038/35140

[48] Thara VK, Fellers JP, Zhou J-M (2003) In planta induced genes of Puccinia triticina. Molecular Plant Pathology 4: 51–56. 10.1046/j.1364-3703.2003.00142.x 20569362

[49] Duplessis S, Cuomo CA, Lin YC, Aerts A, Tisserant E, Veneault-Fourrey C, et al (2011) Obligate biotrophy features unraveled by the genomic analysis of rust fungi. Proc Natl Acad Sci U S A 108: 9166–9171. 10.1073/pnas.1019315108 21536894 PMC3107277

[50] Sanati Nezhad A, Geitmann A (2013) The cellular mechanics of an invasive lifestyle. Journal of Experimental Botany 64: 4709–4728. 10.1093/jxb/ert254 24014865

[51] El Gueddari NE, Rauchhaus U, Moerschbacher BM, Deising HB (2002) Developmentally regulated conversion of surface-exposed chitin to chitosan in cell walls of plant pathogenic fungi. New Phytologist 156: 103–112.

[52] Sohn J, Voegele RT, Mendgen K, Hahn M (2000) High Level Activation of Vitamin B1 Biosynthesis Genes in Haustoria of the Rust Fungus Uromyces fabae. Molecular Plant-Microbe Interactions 13: 629–636. 10830262 10.1094/MPMI.2000.13.6.629

[53] Andreeva L, Heads R, Green CJ (1999) Cyclophilins and their possible role in the stress response. International Journal of Experimental Pathology 80: 305–315. 10632780 10.1046/j.1365-2613.1999.00128.x PMC2517841

[54] Gothel SF, Marahiel MA (1999) Peptidyl-prolyl cis-trans isomerases, a superfamily of ubiquitous folding catalysts. Cell Mol Life Sci 55: 423–436. 10228556 10.1007/s000180050299 PMC11146858

[55] Arevalo-Rodriguez M, Cardenas ME, Wu X, Hanes SD, Heitman J (2000) Cyclophilin A and Ess1 interact with and regulate silencing by the Sin3-Rpd3 histone deacetylase. Embo j 19: 3739–3749. 10899127 10.1093/emboj/19.14.3739 PMC313981

[56] Wang X, Zafian P, Choudhary M, Lawton M (1996) The PR5K receptor protein kinase from Arabidopsis thaliana is structurally related to a family of plant defense proteins. Proceedings of the National Academy of Sciences 93: 2598–2602. 8637920 10.1073/pnas.93.6.2598 PMC39843

[57] Petre B, Major I, Rouhier N, Duplessis S (2011) Genome-wide analysis of eukaryote thaumatin-like proteins (TLPs) with an emphasis on poplar. BMC Plant Biology 11: 33 10.1186/1471-2229-11-33 21324123 PMC3048497

[58] Joly D, Feau N, Tanguay P, Hamelin R (2010) Comparative analysis of secreted protein evolution using expressed sequence tags from four poplar leaf rusts (Melampsora spp.). BMC Genomics 11: 422 10.1186/1471-2164-11-422 20615251 PMC2996950

[59] Saunders DG, Win J, Cano LM, Szabo LJ, Kamoun S, Raffaele S (2012) Using hierarchical clustering of secreted protein families to classify and rank candidate effectors of rust fungi. PLoS One 7: e29847 10.1371/journal.pone.0029847 22238666 PMC3253089

[60] Liu JJ, Sturrock R, Ekramoddoullah AK (2010) The superfamily of thaumatin-like proteins: its origin, evolution, and expression towards biological function. Plant Cell Rep 29: 419–436. 10.1007/s00299-010-0826-8 20204373

[61] Warner JR, McIntosh KB (2009) How common are extraribosomal functions of ribosomal proteins? Mol Cell 34: 3–11. 10.1016/j.molcel.2009.03.006 19362532 PMC2679180

[62] Gao X, Hardwidge PR (2011) Ribosomal protein s3: a multifunctional target of attaching/effacing bacterial pathogens. Front Microbiol 2: 137 10.3389/fmicb.2011.00137 21738525 PMC3125523

[63] Penninckx IAMA, Thomma BPHJ, Buchala A, Métraux J-P, Broekaert WF (1998) Concomitant Activation of Jasmonate and Ethylene Response Pathways Is Required for Induction of a Plant Defensin Gene in Arabidopsis. The Plant Cell Online 10: 2103–2113. 9836748 10.1105/tpc.10.12.2103 PMC143966

[64] Schilmiller AL, Koo AJ, Howe GA (2007) Functional diversification of acyl-coenzyme A oxidases in jasmonic acid biosynthesis and action. Plant Physiol 143: 812–824. 17172287 10.1104/pp.106.092916 PMC1803733

[65] Sohani MM, Schenk PM, Schultz CJ, Schmidt O (2009) Phylogenetic and transcriptional analysis of a strictosidine synthase-like gene family in Arabidopsis thaliana reveals involvement in plant defence responses. Plant Biol (Stuttg) 11: 105–117. 10.1111/j.1438-8677.2008.00139.x 19121120

[66] Pauwels L, Barbero GF, Geerinck J, Tilleman S, Grunewald W, Perez AC, et al (2010) NINJA connects the co-repressor TOPLESS to jasmonate signalling. Nature 464: 788–791. 10.1038/nature08854 20360743 PMC2849182

[67] Moreno JE, Shyu C, Campos ML, Patel LC, Chung HS, Yao J, et al (2013) Negative feedback control of jasmonate signaling by an alternative splice variant of JAZ10. Plant Physiol 162: 1006–1017. 10.1104/pp.113.218164 23632853 PMC3668036

[68] De Lorenzo G, D'Ovidio R, Cervone F (2001) The role of polygalacturonase-inhibiting proteins (PGIPs) in defense against pathogenic fungi. Annu Rev Phytopathol 39: 313–335. 11701868 10.1146/annurev.phyto.39.1.313

[69] JOHNSTON DJ, RAMANATHAN V, WILLIAMSON B (1993) A Protein from Immature Raspberry Fruits which Inhibits Endopolygalacturonases from Botrytis cinerea and other Micro-organisms. Journal of Experimental Botany 44: 971–976.

[70] Salvi G, Giarrizzo F, De Lorenzo G, Cervone F (1990) A Polygalacturonase-Inhibiting Protein in the Flowers of *Phaseolus vulgaris* L. Journal of Plant Physiology 136: 513–518. 10.1104/pp.85.3.631 PMC1054313 16665751

[71] Powell AL, van Kan J, ten Have A, Visser J, Greve LC, Bennett AB, et al (2000) Transgenic expression of pear PGIP in tomato limits fungal colonization. Mol Plant Microbe Interact 13: 942–950. 10975651 10.1094/MPMI.2000.13.9.942

[72] Worberg A, Quandt D, Barniske A-M, Löhne C, Hilu KW, Borsch T (2007) Phylogeny of basal eudicots: insights from non-coding and rapidly evolving DNA. Organisms Diversity & Evolution 7: 55–77.

[73] Van Loon LC, Van Strien EA (1999) The families of pathogenesis-related proteins, their activities, and comparative analysis of PR-1 type proteins. Physiological and Molecular Plant Pathology 55: 85–97.

[74] Bravo JM, Campo S, Murillo I, Coca M, San Segundo B (2003) Fungus- and wound-induced accumulation of mRNA containing a class II chitinase of the pathogenesis-related protein 4 (PR-4) family of maize. Plant Mol Biol 52: 745–759. 13677464 10.1023/a:1025016416951

[75] Duplessis S, Major I, Martin F, Séguin A (2009) Poplar and Pathogen Interactions: Insights fromPopulusGenome-Wide Analyses of Resistance and Defense Gene Families and Gene Expression Profiling. Critical Reviews in Plant Sciences 28: 309–334.

[76] Duo-Chuan L (2006) Review of fungal chitinases. Mycopathologia 161: 345–360. 16761182 10.1007/s11046-006-0024-y

[77] Ghosh M (2006) Antifungal properties of haem peroxidase from Acorus calamus. Annals of botany 98: 1145–1153. 17056613 10.1093/aob/mcl205 PMC2803583

[78] Hammerschmidt R, Kuć J (1982) Lignification as a mechanism for induced systemic resistance in cucumber. Physiological Plant Pathology 20: 61–71.

[79] Peng M, Kuc J (1992) Peroxidase-generated hydrogen peroxide as a source of antifungal activity in vitro and on tobacco leaf disks. Phytopathology 82: 696–699.

[80] Way HM, Kazan K, Goulter KC, Birch RG, Manners JM (2000) Expression of the Shpx2 peroxidase gene of Stylosanthes humilis in transgenic tobacco leads to enhanced resistance to Phytophthora parasitica pv. nicotianae and Cercospora nicotianae. Mol Plant Pathol 1: 223–232. 10.1046/j.1364-3703.2000.00027.x 20572969

[81] Ganz T (2003) Defensins: antimicrobial peptides of innate immunity. Nat Rev Immunol 3: 710–720. 12949495 10.1038/nri1180

[82] Galgóczy L, Kovács L, Vágvölgyi C (2010) Defensin-like antifungal proteins secreted by filamentous fungi. Current Research, Technology and Education Topics in Applied Microbiology and Microbial Technology: 550–559.

[83] Chadha P, Das RH (2006) A pathogenesis related protein, AhPR10 from peanut: an insight of its mode of antifungal activity. Planta 225: 213–222. 16832688 10.1007/s00425-006-0344-7

[84] Lee EJ, Facchini P (2010) Norcoclaurine synthase is a member of the pathogenesis-related 10/Bet v1 protein family. Plant Cell 22: 3489–3503. 10.1105/tpc.110.077958 21037103 PMC2990142

[85] Edreva A (2005) Pathogenesis-related proteins: research progress in the last 15 years. Gen Appl Plant Physiol 31: 105–124.

[86] Rayapuram C, Wu J, Haas C, Baldwin IT (2008) PR-13/Thionin but not PR-1 mediates bacterial resistance in Nicotiana attenuata in nature, and neither influences herbivore resistance. Mol Plant Microbe Interact 21: 988–1000. 10.1094/MPMI-21-7-0988 18533839

[87] Cobbett C, Goldsbrough P (2002) Phytochelatins and metallothioneins: roles in heavy metal detoxification and homeostasis. Annu Rev Plant Biol 53: 159–182. 12221971 10.1146/annurev.arplant.53.100301.135154

[88] Wong HL, Sakamoto T, Kawasaki T, Umemura K, Shimamoto K (2004) Down-Regulation of Metallothionein, a Reactive Oxygen Scavenger, by the Small GTPase OsRac1 in Rice. Plant Physiology 135: 1447–1456. 15220467 10.1104/pp.103.036384 PMC519061

[89] Grennan AK (2011) Metallothioneins, a diverse protein family. Plant Physiol 155: 1750–1751. 10.1104/pp.111.900407 21459979 PMC3091091

[90] Nishimura S, Tatano S, Miyamoto Y, Ohtani K, Fukumoto T, Gomi K, et al (2013) A zinc-binding citrus protein metallothionein can act as a plant defense factor by controlling host-selective ACR-toxin production. Plant Mol Biol 81: 1–11. 10.1007/s11103-012-9976-0 23086497

[91] Tremblay A, Hosseini P, Alkharouf NW, Li S, Matthews BF (2011) Gene Expression in Leaves of Susceptible Glycine max during Infection with Phakopsora pachyrhizi Using Next Generation Sequencing. Sequencing 2011: 1–14. 21808638

[92] Mahomed W, van den Berg N (2011) EST sequencing and gene expression profiling of defence-related genes from Persea americana infected with Phytophthora cinnamomi. BMC Plant Biology 11: 167 10.1186/1471-2229-11-167 22108245 PMC3233532

[93] Coram TE, Wang M, Chen X (2008) Transcriptome analysis of the wheat-Puccinia striiformis f. sp. tritici interaction. Mol Plant Pathol 9: 157–169. 10.1111/j.1364-3703.2007.00453.x 18705849 PMC6640478

[94] Kariola T, Brader G, Helenius E, Li J, Heino P, Palva ET (2006) EARLY RESPONSIVE TO DEHYDRATION 15, a negative regulator of abscisic acid responses in Arabidopsis. Plant Physiol 142: 1559–1573. 17056758 10.1104/pp.106.086223 PMC1676049

[95] Prescott AG, Stamford NP, Wheeler G, Firmin JL (2002) In vitro properties of a recombinant flavonol synthase from Arabidopsis thaliana. Phytochemistry 60: 589–593. 12126705 10.1016/s0031-9422(02)00155-3

[96] Lukacin R, Wellmann F, Britsch L, Martens S, Matern U (2003) Flavonol synthase from Citrus unshiu is a bifunctional dioxygenase. Phytochemistry 62: 287–292. 12620339 10.1016/s0031-9422(02)00567-8

[97] Treutter D (2005) Significance of flavonoids in plant resistance and enhancement of their biosynthesis. Plant Biol (Stuttg) 7: 581–591. 16388461 10.1055/s-2005-873009

[98] Maimbo M, Ohnishi K, Hikichi Y, Yoshioka H, Kiba A (2007) Induction of a small heat shock protein and its functional roles in Nicotiana plants in the defense response against Ralstonia solanacearum. Plant Physiol 145: 1588–1599. 17965181 10.1104/pp.107.105353 PMC2151688

[99] Petriccione M, Di Cecco I, Arena S, Scaloni A, Scortichini M (2013) Proteomic changes in Actinidia chinensis shoot during systemic infection with a pandemic Pseudomonas syringae pv. actinidiae strain. J Proteomics 78: 461–476. 10.1016/j.jprot.2012.10.014 23099348

[100] Kanzaki H, Saitoh H, Ito A, Fujisawa S, Kamoun S, Katou S, et al (2003) Cytosolic HSP90 and HSP70 are essential components of INF1-mediated hypersensitive response and non-host resistance to Pseudomonas cichorii in Nicotiana benthamiana. Mol Plant Pathol 4: 383–391. 10.1046/j.1364-3703.2003.00186.x 20569398

[101] Hubert DA, Tornero P, Belkhadir Y, Krishna P, Takahashi A, Shirasu K, et al (2003) Cytosolic HSP90 associates with and modulates the Arabidopsis RPM1 disease resistance protein. Embo j 22: 5679–5689. 14592967 10.1093/emboj/cdg547 PMC275404

[102] Schulze-Lefert P (2004) Plant Immunity: The Origami of Receptor Activation. Current Biology 14: R22–R24. 14711430

[103] Kader JC (1996) LIPID-TRANSFER PROTEINS IN PLANTS. Annu Rev Plant Physiol Plant Mol Biol 47: 627–654. 15012303 10.1146/annurev.arplant.47.1.627

[104] Collins NC, Thordal-Christensen H, Lipka V, Bau S, Kombrink E, Qiu JL, et al (2003) SNARE-protein-mediated disease resistance at the plant cell wall. Nature 425: 973–977. 14586469 10.1038/nature02076

[105] Lipka V, Kwon C, Panstruga R (2007) SNARE-ware: the role of SNARE-domain proteins in plant biology. Annu Rev Cell Dev Biol 23: 147–174. 17506694 10.1146/annurev.cellbio.23.090506.123529

[106] Wald FA, Kissen R, du Jardin P, Moreno S (2003) Characterization of UDP-glucose:protein transglucosylase genes from potato. Plant Mol Biol 52: 705–714. 13677461 10.1023/a:1025061324856

[107] Polesani M, Desario F, Ferrarini A, Zamboni A, Pezzotti M, Kortekamp A, et al (2008) cDNA-AFLP analysis of plant and pathogen genes expressed in grapevine infected with Plasmopara viticola. BMC Genomics 9: 142 10.1186/1471-2164-9-142 18366764 PMC2292706

[108] Minic Z, Jouanin L (2006) Plant glycoside hydrolases involved in cell wall polysaccharide degradation. Plant Physiol Biochem 44: 435–449. 17023165 10.1016/j.plaphy.2006.08.001

[109] Singh A, Prasad R (2009) Salt stress affects growth and cell wall bound enzymes in Arachis hypogaea L. seedlings. International Journal of Integrative Biology 7: 117–123.

[110] Hernandez-Blanco C, Feng DX, Hu J, Sanchez-Vallet A, Deslandes L, Llorente F, et al (2007) Impairment of cellulose synthases required for Arabidopsis secondary cell wall formation enhances disease resistance. Plant Cell 19: 890–903. 17351116 10.1105/tpc.106.048058 PMC1867366

[111] Rose JK, Braam J, Fry SC, Nishitani K (2002) The XTH family of enzymes involved in xyloglucan endotransglucosylation and endohydrolysis: current perspectives and a new unifying nomenclature. Plant Cell Physiol 43: 1421–1435. 12514239 10.1093/pcp/pcf171

[112] Gruner K, Griebel T, Navarova H, Attaran E, Zeier J (2013) Reprogramming of plants during systemic acquired resistance. Front Plant Sci 4: 252 10.3389/fpls.2013.00252 23874348 PMC3711057

[113] Luo B, Xue XY, Hu WL, Wang LJ, Chen XY (2007) An ABC transporter gene of Arabidopsis thaliana, AtWBC11, is involved in cuticle development and prevention of organ fusion. Plant Cell Physiol 48: 1790–1802. 17989085 10.1093/pcp/pcm152

[114] Krattinger SG, Lagudah ES, Spielmeyer W, Singh RP, Huerta-Espino J, McFadden H, et al (2009) A putative ABC transporter confers durable resistance to multiple fungal pathogens in wheat. Science 323: 1360–1363. 10.1126/science.1166453 19229000

[115] Yu X, Wang X, Wang C, Chen X, Qu Z, Yu X, et al (2010) Wheat defense genes in fungal (Puccinia striiformis) infection. Funct Integr Genomics 10: 227–239. 10.1007/s10142-010-0161-8 20186453

[116] Peumans WJ, Van Damme EJ (1995) Lectins as plant defense proteins. Plant Physiol 109: 347–352. 7480335 10.1104/pp.109.2.347 PMC157596

[117] Knepper C, Savory EA, Day B (2011) Arabidopsis NDR1 is an integrin-like protein with a role in fluid loss and plasma membrane-cell wall adhesion. Plant Physiol 156: 286–300. 10.1104/pp.110.169656 21398259 PMC3091050

[118] Goritschnig S, Krasileva KV, Dahlbeck D, Staskawicz BJ (2012) Computational Prediction and Molecular Characterization of an Oomycete Effector and the Cognate *Arabidopsis* Resistance Gene. PLoS Genet 8: e1002502 10.1371/journal.pgen.1002502 22359513 PMC3280963

[119] Zheng MS, Takahashi H, Miyazaki A, Hamamoto H, Shah J, Yamaguchi I, et al (2004) Up-regulation of Arabidopsis thaliana NHL10 in the hypersensitive response to Cucumber mosaic virus infection and in senescing leaves is controlled by signalling pathways that differ in salicylate involvement. Planta 218: 740–750. 14666423 10.1007/s00425-003-1169-2

[120] Cacas JL, Petitot AS, Bernier L, Estevan J, Conejero G, Mongrand S, et al (2011) Identification and characterization of the Non-race specific Disease Resistance 1 (NDR1) orthologous protein in coffee. BMC Plant Biol 11: 144 10.1186/1471-2229-11-144 22023696 PMC3212813

[121] Yanhui C, Xiaoyuan Y, Kun H, Meihua L, Jigang L, Zhaofeng G, et al (2006) The MYB transcription factor superfamily of Arabidopsis: expression analysis and phylogenetic comparison with the rice MYB family. Plant Mol Biol 60: 107–124. 16463103 10.1007/s11103-005-2910-y

[122] El-kereamy A, Jayasankar S (2013) Cloning and differential expression of a plum single repeat-MYB, PdMYB3, in compatible and incompatible interactions during fungal infection. Canadian Journal of Plant Science 93: 599–605.

[123] Fukunaga K, Arita M, Takahashi M, Morris AJ, Pfeffer M, Levy BD (2006) Identification and functional characterization of a presqualene diphosphate phosphatase. J Biol Chem 281: 9490–9497. 16464866 10.1074/jbc.M512970200

[124] Rasbery JM, Shan H, LeClair RJ, Norman M, Matsuda SP, Bartel B (2007) Arabidopsis thaliana squalene epoxidase 1 is essential for root and seed development. J Biol Chem 282: 17002–17013. 17426032 10.1074/jbc.M611831200

[125] Dean JV, Mohammed LA, Fitzpatrick T (2005) The formation, vacuolar localization, and tonoplast transport of salicylic acid glucose conjugates in tobacco cell suspension cultures. Planta 221: 287–296. 15871031 10.1007/s00425-004-1430-3

[126] Loake G, Grant M (2007) Salicylic acid in plant defence—the players and protagonists. Current Opinion in Plant Biology 10: 466–472. 17904410 10.1016/j.pbi.2007.08.008

[127] Park HJ, Woo JY, Kim YJ (2011) Suppression of UDP-glycosyltransferase-coding Arabidopsis thaliana UGT74E2 gene expression leads to increased resistance to Psuedomonas syringae pv. tomato DC3000 infection. The Plant Pathology Journal 27: 170–182.

[128] Karim S, Holmstrom KO, Mandal A, Dahl P, Hohmann S, Brader G, et al (2007) AtPTR3, a wound-induced peptide transporter needed for defence against virulent bacterial pathogens in Arabidopsis. Planta 225: 1431–1445. 17143616 10.1007/s00425-006-0451-5

[129] Yamamoto H, Katano N, Ooi A, Inoue K (2000) Secologanin synthase which catalyzes the oxidative cleavage of loganin into secologanin is a cytochrome P450. Phytochemistry 53: 7–12. 10656401 10.1016/s0031-9422(99)00471-9

[130] Facchini PJ (2001) ALKALOID BIOSYNTHESIS IN PLANTS: Biochemistry, Cell Biology, Molecular Regulation, and Metabolic Engineering Applications. Annu Rev Plant Physiol Plant Mol Biol 52: 29–66. 11337391 10.1146/annurev.arplant.52.1.29

[131] Dodds PN, Rafiqi M, Gan PHP, Hardham AR, Jones DA, Ellis JG (2009) Effectors of biotrophic fungi and oomycetes: pathogenicity factors and triggers of host resistance. New Phytologist 183: 993–1000. 10.1111/j.1469-8137.2009.02922.x 19558422

[132] Ryals JA, Neuenschwander UH, Willits MG, Molina A, Steiner HY, Hunt MD (1996) Systemic Acquired Resistance. The Plant Cell Online 8: 1809–1819. 10.1105/tpc.8.10.1809 PMC161316 12239363

[133] Marroni F, Pinosio S, Di Centa E, Jurman I, Boerjan W, Felice N, et al (2011) Large‐scale detection of rare variants via pooled multiplexed next‐generation sequencing: towards next‐generation Ecotilling. The Plant Journal 67: 736–745. 10.1111/j.1365-313X.2011.04627.x 21554453

[134] Herrera-Estrella L, Depicker A, Van Montagu M, Schell J (1983) Expression of chimaeric genes transferred into plant cells using a Ti-plasmid-derived vector. Nature 303: 209–213. 1422044

[135] Wulff BB, Horvath DM, Ward ER (2011) Improving immunity in crops: new tactics in an old game. Curr Opin Plant Biol 14: 468–476. 10.1016/j.pbi.2011.04.002 21531167

[136] Farnham G, Baulcombe DC (2006) Artificial evolution extends the spectrum of viruses that are targeted by a disease-resistance gene from potato. Proceedings of the National Academy of Sciences 103: 18828–18833. 17021014 10.1073/pnas.0605777103 PMC1693747

[137] Wang W, Lu A-M, Ren Y, Endress ME, Chen Z-D (2009) Phylogeny and classification of Ranunculales: evidence from four molecular loci and morphological data. Perspectives in Plant Ecology, Evolution and Systematics 11: 81–110.

