# Supplementary figures and images for: The Effect on the Transcriptome of Anemone coronaria following Infection with Rust (Tranzschelia discolor)
Source: PLoS One. 2015 Mar 13;10(3):e0118565. doi: 10.1371/journal.pone.0118565 (PMC4359109; doi:10.1371/journal.pone.0118565)

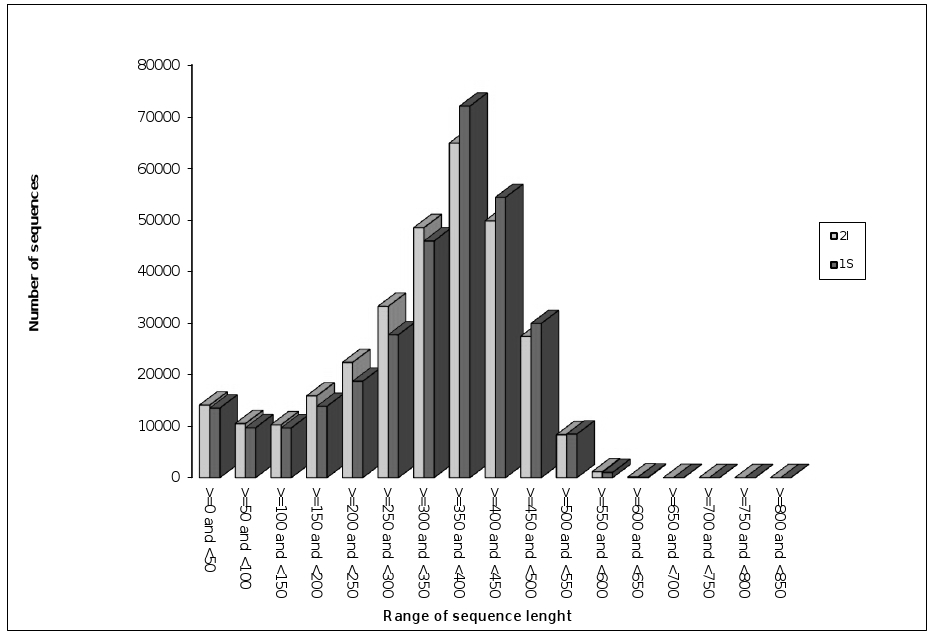

Supplement: S1 Fig — 2I represents infected library and 1S represent uninfected library. (TIF) [file pone.0118565.s003.tif]

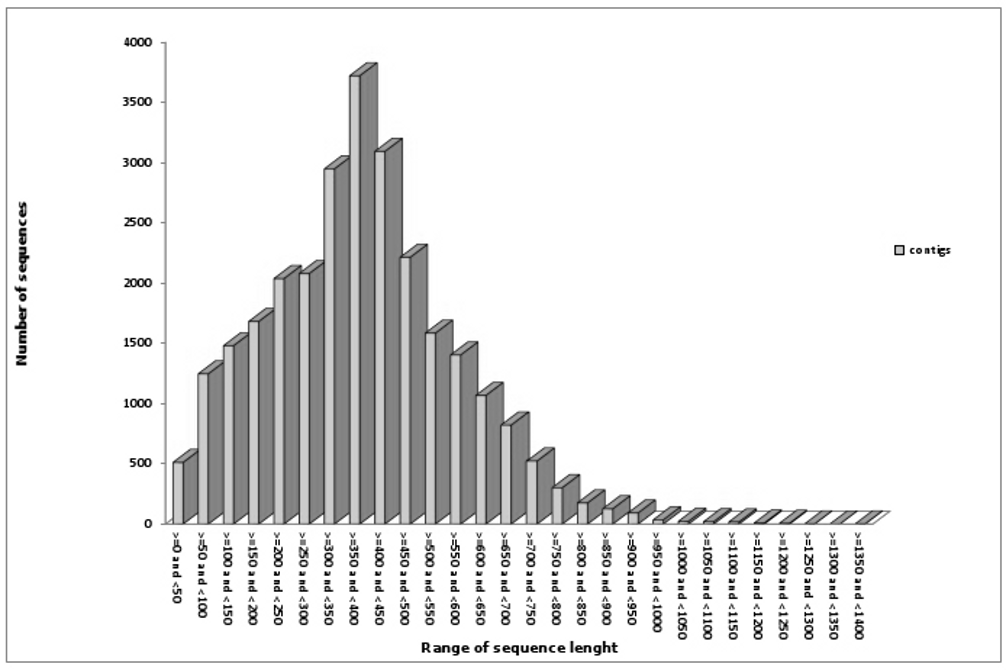

Supplement: S2 Fig — The contigs were mass assembled from the two libraries; the mean length is 377 nt. (TIF) [file pone.0118565.s004.tif]

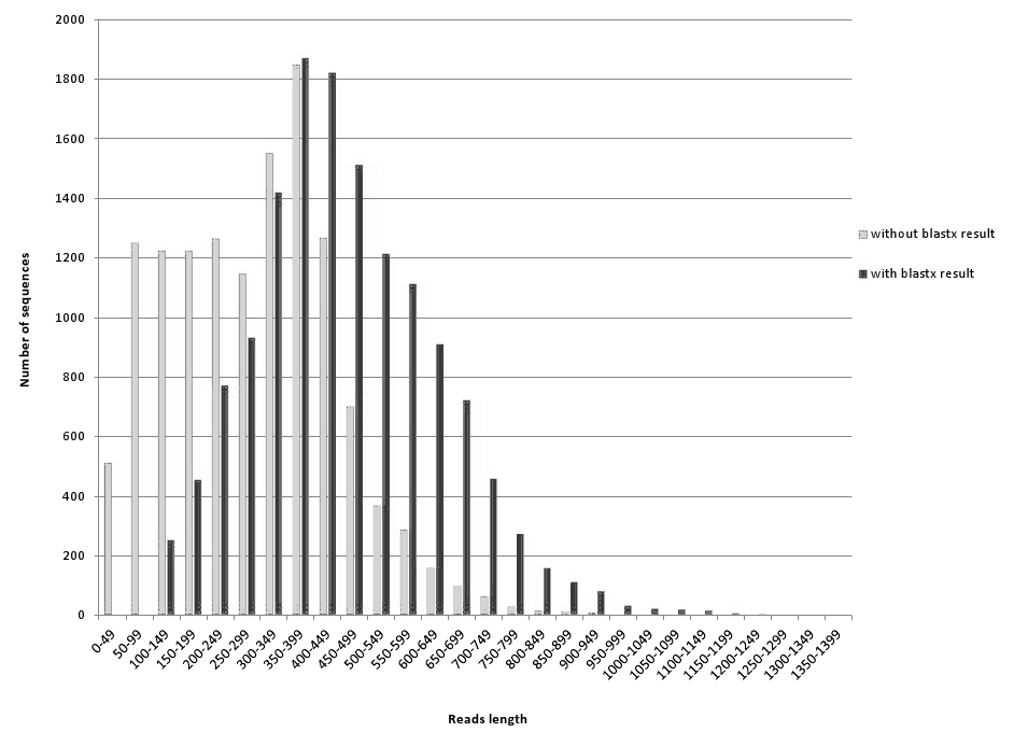

Supplement: S3 Fig — The 50.5% of the predicted translation products shared significant homology with known protein sequences deposited in GenBank and 1.7% with hypothetical proteins, leaving 47.8% of the sequences unannotated. The proportion of sequences lacking any BLASTx alignment and shorter than 250 nt was 42.1%. (TIF) [file pone.0118565.s005.tif]

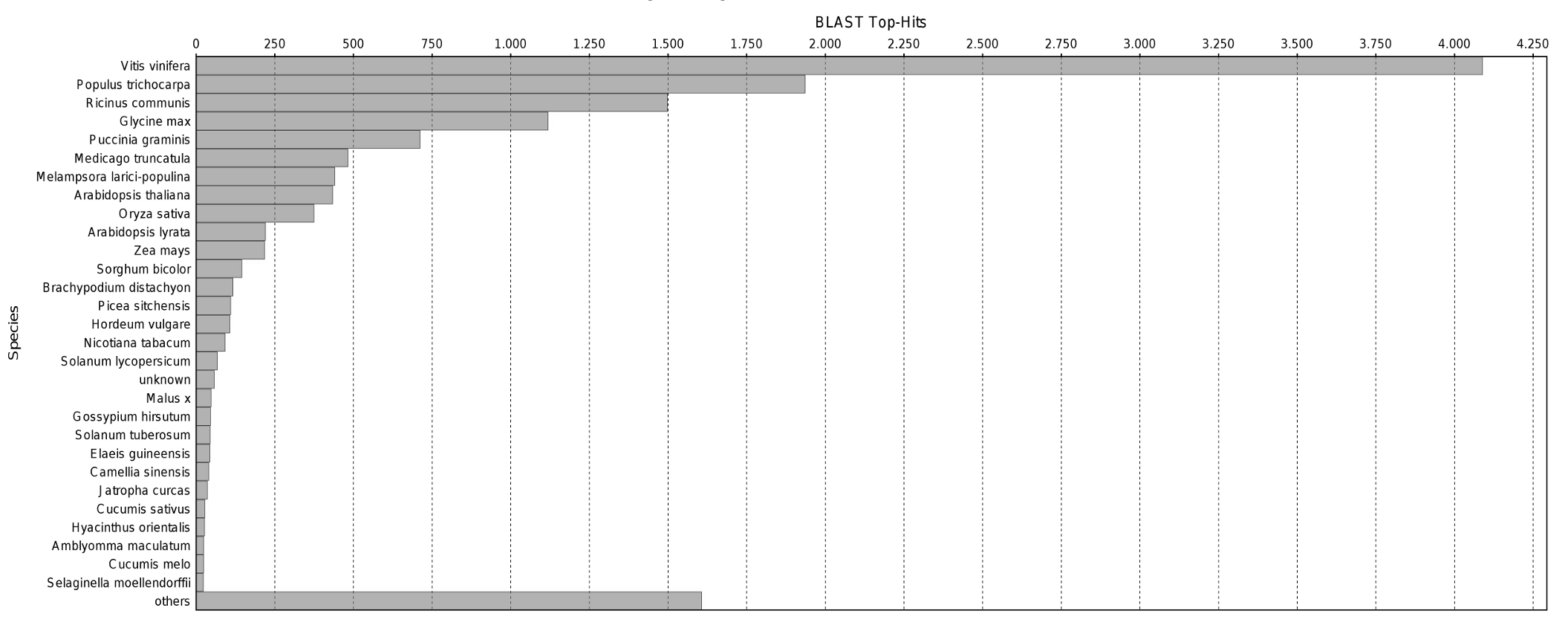

Supplement: S4 Fig — Vitis vinifera (grape) is the most frequently occurring species, followed by Populus tricocarpa (black cottonwood), Ricinus communis (the castor oil plant), Glycine max (soybean) and Puccinia graminis (cereal stem black rust). (TIF) [file pone.0118565.s006.tif]
